# Variational quantum enhanced deep transfer learning for small underwater aqua species image classification

**DOI:** 10.1038/s41598-025-22524-y

**Published:** 2025-11-04

**Authors:** Sugunapriya A, Markkandan S

**Affiliations:** Vellore Institute of Technology, Chennai, 600127 India

**Keywords:** Aquaculture, Classification, Deep learning, Underwater, Quantum learning, Variational quantum circuit, Environmental impact, Electrical and electronic engineering, Marine biology

## Abstract

Precise underwater classification of small aquaculture species is essential for sustainable fisheries management, biodiversity monitoring, and automated marine ecosystem analysis. But it is still a challenging task owing to underwater image distortions from poor visibility, lighting changes, occlusions, and the high computational complexity of traditional deep learning models. To address these issues, we propose a Lightweight Variational Quantum Enhanced Deep Transfer Learning framework. This hybrid deep transfer learning model integrates pretrained classical convolutional neural networks with variational quantum circuits to improve feature representation and classification efficiency. The framework is designed to reduce computational complexity while enhancing accuracy by leveraging quantum feature extraction techniques. Experimental evaluations on curated small aquafarming species dataset demonstrate that the proposed approach achieves high classification accuracy (up to 99.25%) with significantly fewer parameters and floating-point operations, indicating its potential for resource-constrained applications. Ablation studies further validate the impact of quantum layers on model performance. These results suggest that quantum deep transfer learning models can offer a promising direction for robust and efficient underwater species classification.

## Introduction

The global aquaculture industry plays a vital role in meeting the increasing demand for seafood while contributing to food security and economic growth. Among aquatic species, shrimp and prawn culture are particularly important due to their high commercial value^1^. Accurate monitoring and classification of these species are essential for sustainable farming practices, disease prevention, and maximizing yield. Small aquatic species such as larvae, prawns, and shrimp require precise classification to support biodiversity monitoring, health assessment, and quality evaluation. Beyond their ecological role, these species underpin a billion-dollar industry in which correct discrimination directly influences market categorization and pricing.

Despite its importance, small-species classification in aquaculture remains highly challenging. Species often range from less than 1 mm to 300 mm, display subtle morphological differences, and appear with low contrast against underwater backgrounds. Variations in illumination, water turbidity, suspended particles, and occlusion further complicate recognition. Manual identification is labor-intensive, error-prone, and impractical for large-scale monitoring. These challenges underscore the need for automated, reliable, and real-time classification solutions.

Artificial intelligence (AI) and deep learning (DL) approaches have shown promise in addressing these issues. Convolutional neural networks (CNNs), generative adversarial networks (GANs), and augmentation strategies have been applied to improve underwater image classification. While effective in certain contexts, these models face limitations. CNN-based architectures require large labeled datasets and substantial computational resources, limiting their suitability for deployment on resource-constrained platforms such as underwater drones. Their performance often degrades when applied to unseen underwater environments with varying water quality or lighting. Moreover, conventional CNNs focus primarily on local spatial features and struggle to capture long-range dependencies, reducing their ability to differentiate between morphologically similar species.

To overcome these barriers, researchers have begun exploring quantum computing (QC) as a transformative paradigm for machine learning. Unlike classical computers that process binary states, quantum computers leverage qubits in superposition, enabling parallel computation and efficient handling of complex data. Quantum machine learning (QML) integrates QC principles into deep learning models, offering several potential advantages: (i) improved feature representation through high-dimensional data encoding, (ii) enhanced efficiency using quantum support vector machines (QSVMs) and quantum neural networks (QNNs), (iii) increased robustness to noisy data by exploiting entanglement and error correction, and (iv) the development of hybrid quantum–classical models such as quantum convolutional neural networks (QCNNs). Recent studies suggest that quantum-enhanced methods can significantly benefit aquaculture applications, including shrimp detection, disease identification, and biomass estimation. Despite these advances, existing research in aquaculture imaging and QML still presents critical gaps. To contextualize the proposed VQEDTL framework, this section reviews prior work, Table 1, along four main dimensions: (i) datasets and image acquisition, (ii Deep Learning for Shrimp Recognition and Classification, (iii) Detection, Localization, and Biomass Estimation, and (iv) hybrid classical–quantum approaches.

**Table 1:** Literature survey on existing work in underwater aquafarming species classification.

Work	Model type	Core innovation	Application scenario	Limitations	Differences from VQEDTL
Sun et al.^2^	Pure Quantum CNN (SQ-CNN)	Proposes a scalable QCNN architecture with reduced circuit depth for large-scale image classification	Generic image classification (benchmark datasets)	Not domain-specific; lacks hybrid CNN integration; challenges in direct underwater adaptation	VQEDTL leverages classical CNNs for domain-specific feature extraction and applies variational encoding at the feature level, making it more practical for underwater aquaculture images
Melinda et al.^3^	CNN	Vannamei shrimp classification	Shrimp aquaculture	Limited handling of occlusion, small species	Encodes subtle morphological patterns via quantum feature embedding
Easom-McCaldin et al.^5^	Single-Qubit Encoding	Efficient quantum image classification	General image datasets	Limited scalability, not aquaculture-specific	Scales hybrid quantum layers for real-world aquaculture datasets
Trochun and Gordienko^6^	Hybrid QCNN / Quanvolutional	Hybrid quantum–classical image classification	General image datasets	Limited application to aquaculture	Applies hybrid QC specifically to shrimp/prawn recognition
Warrier et al.^7^	Hybrid QCNN	On-board edge classification	Underwater species	Not focused on small species	Explicitly tuned for small shrimp/prawn in complex environments
Pravin et al.^8^	Hybrid Classical–Quantum	Marine animal identification	Biodiversity monitoring	General species, no small species focus	Designed for small shrimp/prawn with robustness to occlusion and low-light
Zariful et al.^9^	CNN	Deep learning for prawn recognition	Challenging aquaculture environments	High computation, small species not fully addressed	Adds quantum layers for efficient feature representation and better handling of small species
Satoto et al.^10^	CNN + Data Augmentation	Coastal shrimp classification	Coastal shrimp species	Limited robustness to occlusion and low light	Extends robustness to low-light, turbidity, occlusion, and small species
Huang et al.^11^	Hybrid QCNN	Robust image classification	Adversarial/noisy images	Not focused on small underwater species	Optimized for small species under occlusion and low-light
Fauzi et al.^12^	CNN + Image Processing	Low-light image augmentation	Low-light underwater shrimp images	Overfitting, computationally expensive	Combines classical and quantum layers for efficient, robust low-light recognition
Qin et al.^13^	Dense Unbiased Teacher	Semi-supervised shrimp detection	Shrimp aquaculture	Limited to detection, not classification	Performs both detection and classification with quantum-enhanced features
Wang et al.^14^	VQDNN	Variational quantum deep networks	General image recognition	Not aquaculture-specific	Adapts QML for aquaculture-specific small species recognition
Suárez et al.^15^	CNN	Automated shrimp classification	Coastal and aquaculture species	Not robust to low-light or turbid water	Explicitly addresses low-light and turbidity challenges
Li et al.^16^	Hybrid QNN	Quantum deep convolutional networks	Image recognition	Not tailored to small species or underwater conditions	Targets underwater shrimp/prawn recognition under challenging conditions
Hu et al.^17^	CNN (ShrimpNet)	Automated shrimp recognition	Shrimp species	Requires large labeled datasets	Reduces dataset dependency with hybrid quantum encoding

### Datasets and image acquisition

The availability of well-annotated datasets has significantly accelerated progress in aquaculture imaging research. Ramírez-Coronel et al.^18^ introduced a dataset of Litopenaeus vannamei shrimp for biomass estimation and organism detection, providing an essential benchmark for supervised learning tasks. Similarly, Melinda et al.^3^ constructed an image dataset from Vannamei shrimp cultivation, emphasizing acquisition and preprocessing procedures to facilitate accurate classification. Zhou et al.^19^ extended this trend by developing a dataset for shrimp larvae, applying density map regression to count, locate, and size larvae with high precision. These works highlight the necessity of specialized datasets that capture underwater challenges such as occlusion, turbidity, and low-light conditions.

### Deep learning for shrimp recognition and classification

Deep learning (DL) has demonstrated strong performance in aquaculture image analysis, particularly for small species such as prawn and shrimp. Zariful et al.^9^ analyzed DL algorithms for prawn aquaculture under challenging environments, showing the robustness of CNN-based architectures. Satoto et al.^10^ employed InceptionResNetV2 with extensive data augmentation for coastal shrimp species classification, demonstrating the effectiveness of transfer learning. Hu et al.^17^ proposed ShrimpNet, a CNN optimized for shrimp recognition, while Suárez et al.^15^ extended DL pipelines for automated shrimp classification. These studies collectively indicate that transfer learning and strong backbone networks generally outperform handcrafted or domain-specific approaches, particularly under data limitations.

### Detection, localization, and biomass estimation

Shrimp detection and localization are critical for biomass monitoring and real-time aquaculture management. Tang et al.^20^ developed MGC-YOLO, an underwater biomimetic shrimp detector capable of distinguishing shrimp from foreign objects. Qin et al.^13^ introduced a semi-supervised Dense Unbiased Teacher model to leverage unlabeled data for improved shrimp detection. Zhou et al.^19^ applied density map regression for shrimp larvae to enable precise population counting and sizing. These studies suggest that YOLO-based methods are effective for real-time monitoring, while regression-based approaches are advantageous for dense populations and larval estimation.

### Data augmentation and low-light imaging

Underwater environments often suffer from poor image quality, particularly in low-light conditions. Fauzi et al.^12^ proposed image processing–based augmentation strategies to enhance CNN performance on low-light aquaculture images. Mohan Krishnan et al.^21^ further combined YOLO-based detection with diverse augmentation methods to improve shrimp disease classification in variable conditions. These findings underscore the importance of augmentation and preprocessing techniques for robust aquaculture image analysis.

### Shrimp disease detection and health monitoring

Disease detection remains one of the most important applications of AI in aquaculture. Varma and Krishna^22^ developed SDNet, which integrates unsupervised learning with deep CNNs to address limited disease-label availability. Mohan Krishnan et al.^21^ enhanced disease detection using YOLO with augmentation, while Warrier et al.^7^ proposed on-board classification of underwater images using a hybrid classical–quantum CNN, optimized for deployment on resource-constrained devices. These works collectively emphasize that both annotation-efficient learning and lightweight architectures are key for practical disease monitoring.

### Hybrid classical–quantum approaches

Quantum machine learning (QML) has emerged as a promising direction for addressing computational efficiency and parameter overhead in DL. Wang et al.^14^ introduced variational quantum deep neural networks (VQDNNs), while Li et al.^16^ proposed a quantum deep CNN for image recognition. Zhao et al.^23^ designed QDNNs with embedded quantum layers, and Kim et al.^24^ demonstrated classical-to-quantum transfer learning for improved generalization. Warrier et al.^7^ deployed a hybrid CNN–QNN for on-board underwater image classification, and Pravin et al.^8^ introduced hybrid algorithms for marine animal identification. Other works^2,4–6,11,25–32^ surveyed QML mechanisms and highlighted their potential for parameter-efficient, scalable, and noise-robust classification. Collectively, these studies indicate that hybrid classical–quantum approaches can enhance expressive power, reduce training costs, and enable edge-device deployment in aquaculture monitoring.

### Reviews and research trends

Several surveys have synthesized advancements in aquaculture AI and QML. Sun et al.^33^ provided a comprehensive review of DL methods in aquaculture, emphasizing applications in biomass estimation, disease detection, and environmental monitoring. Fernandes and DMello^34^ analyzed AI adoption in aquaculture, highlighting challenges and opportunities for integrating DL and QML in real-time monitoring. In the quantum domain, Alchieri et al.^25^, Priyanka et al.^27^, Tychola et al.^26^, and Mahargya et al.^32^ systematically reviewed quantum-enhanced computer vision, object detection, and recognition, identifying research trends and open challenges. These reviews consistently underline the scarcity of benchmark datasets, the difficulty of transferring models to real-world conditions, and the lack of standardized evaluation protocols.

### Research gaps and future directions

Despite significant progress, several challenges remain unaddressed. First, the absence of standardized benchmark datasets limits comparability across studies. Second, variability in lighting, turbidity, and occlusion necessitates domain adaptation strategies. Third, lightweight, interpretable, and annotation-efficient models are essential for disease monitoring in aquaculture settings. Finally, although hybrid classical–quantum models show promise for reducing computational complexity and improving generalization, their practical benefits in large-scale aquaculture deployment remain largely unproven. Addressing these gaps will be crucial for developing scalable, reliable, and resource-efficient intelligent aquaculture systems.

As illustrated in Table 1, existing CNN-based and hybrid classical–quantum approaches face challenges in small-species recognition, low-light environments, occlusion, or high computational overhead. The proposed VQEDTL framework addresses the limitations of existing hybrid approaches through a structured integration of classical and quantum components. Unlike prior models that apply quantum circuits directly to raw images or large feature maps, VQEDTL applies variational quantum encoding to compact CNN-extracted feature vectors. This architectural choice minimizes quantum overhead while enabling the quantum layers to capture high-dimensional correlations and long-range dependencies that CNNs alone struggle to model in low-contrast and noisy underwater environments. The innovation of VQEDTL lies in its dual-transfer learning design: pretrained model is used to transfer classical visual knowledge for robust local feature extraction, while a variational quantum circuit simultaneously transfers quantum representational capacity to capture global dependencies. A feature-fusion mechanism integrates classical features embeddings with variational quantum embeddings, preserving complementary information that dense classical projections typically discard. VQEDTL combines deep transfer learning with variational quantum layers in a carefully designed 4-qubit circuit using angle embedding and strongly entangling layers. This design minimizes quantum overhead while enhancing feature separability in a high-dimensional space. By focusing on architectural innovation and the mechanism by which quantum encoding augments classical feature representation, the framework clearly distinguishes itself from prior hybrid and classical approaches.

The main contribution of the work is,

- Proposed VQEDTL, that combines classical feature extraction (CFE) layer with quantum feature extraction (QFE) layer to enhance classification performance by improving feature separability in high-dimensional space. The novel idea is integrating angle embedding and strongly entangling layers to encode and transform classical features, that has reduced informative features into a 4-qubit quantum circuit, minimizes quantum overhead while leveraging its strengths.
- A multiclass dataset has been curated by collecting images of five different classes of prawn and shrimp images from available source and previously published research, ensuring diversity and robustness in training data.
- Evaluated the effectiveness of proposed VQEDTL models in comparison to SOTA, state of the art models which attains improvements in accuracy, computational efficiency, and robustness in real-world underwater aquaculture conditions.
- Performed ablation study on how increasing the number of entanglement layers in the quantum circuit affects performance metrics accuracy, precision, recall, and F1-score of the proposed model.

The remaining of the letter includes, Section II. Dataset, Section III. Building Q layer, Section IV. Method, Section V. Result and Discussion, Section VI. Conclusion and Future work.

## Methods

### Dataset curation and pre-processing

**Fig. 1:**
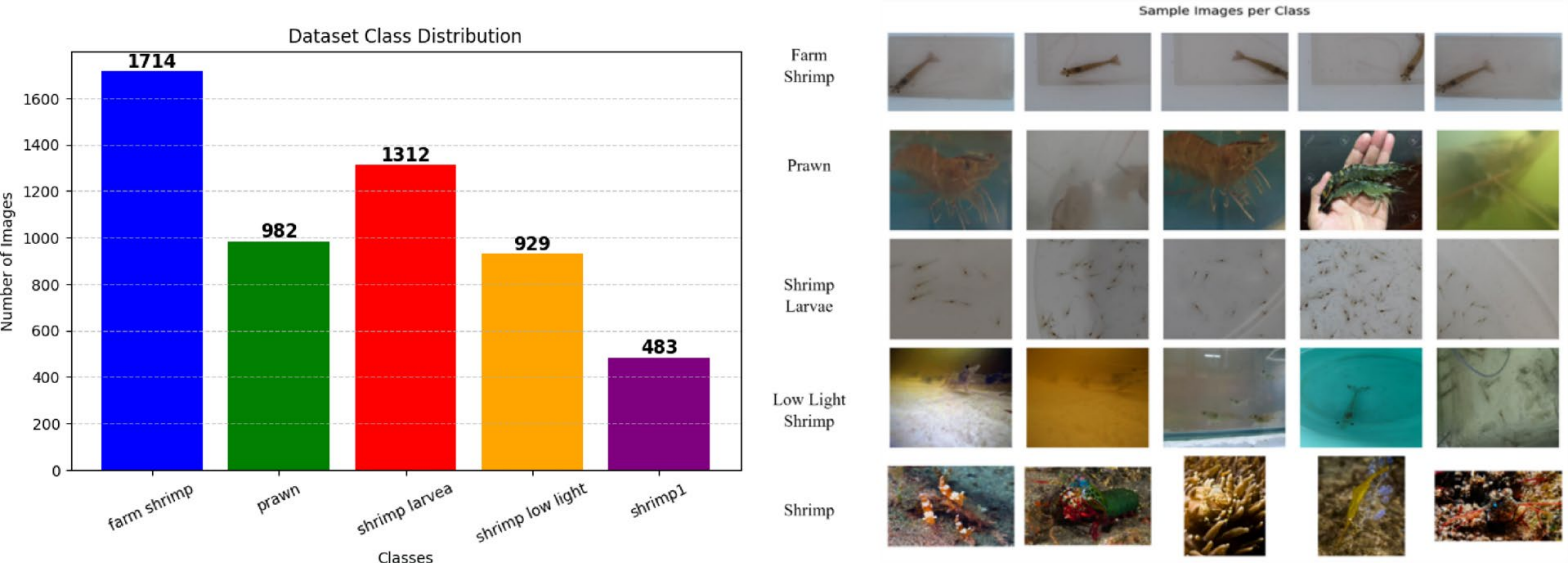
Class distribution of curated shrimp and prawn dataset.

In this proposed work, we utilized a comprehensive dataset of small underwater species consisting of five distinct classes: farm shrimp^18^, shrimp larvae, prawn, shrimp in low light, and shrimp1. Due to their small size, shrimp and prawn require higher-resolution imaging to capture fine details. Their body structures, such as antennae, legs, and segmented bodies, are delicate and visually complex, making classification more challenging. The dataset includes larvae and low-light conditions, further reinforcing the focus on small-scale species, where visibility and size variation play a crucial role in classification. The dataset is prepared by collecting each available different classes of dataset from available net source and also from earlier work. Five selective classes of dataset, Fig. 1 are framed under common folder to perform classification training of the model. The dataset comprises of total 5420 images, systematically splitted into training, validation, and test sets in a 70-20-10 ratio to ensure balanced and representative data distribution.

To maintain a balanced class distribution across different subsets of the dataset, we performed a stratified split of the data and the split was carried out in the way which ensures that the class proportions remain consistent in each subset. the dataset was split into, Train set: 70% of the data (3793 images), Validation set: 20% of the remaining data (1090 images), Test set: 10% of the remaining data (537 images). To improve model generalization and robustness, particularly in the context of varying underwater imaging conditions, image augmentation was performed on the training dataset. This technique synthetically increased the size of the dataset by applying random transformations to the images. We leveraged TensorFlow’s ImageDataGenerator to implement the following augmentations: Rotation (± 30°), Width & Height Shifts (± 20%), Shear & Zoom (± 20%), Horizontal Flipping and Fill Mode (‘nearest’). In addition to augmentation, the images were resized to a standard 224 × 224 pixels to match the input dimensions expected by the deep learning model. Normalization was also performed using the pre trained CNN preprocessing function, which rescale pixel to the required range for compatibility with a pre-trained model. This preprocessing step aligns the dataset with the expectations of the model, enhancing the overall training process.

### Building quantum layer, Ql

Quantum Computing, QC^35,36^ is an emerging discipline that leverages the principles of quantum physics to conduct computations impossible for traditional computers Unlike conventional computing, which uses binary bits to execute information, quantum computers use quantum bits (qubits) that simultaneously exist in a superposition of both states. This ability allows quantum computers to do numerous calculations simultaneously, greatly increasing the efficiency of some calculations. The superposition principle is a basic principle of QC since a qubit is in both ∣0⟩ and ∣1⟩ states at the same time as well as in the combined state ∣0⟩ and ∣1⟩, ∣ψ⟩ = α|0⟩ + β|1⟩, ∣ψ⟩ is a single qubit, ket 0 (∣0⟩) is the 0 state and ket 1 (∣1⟩) is the 1 state and α and β are complex parameters such that |α|^2^ +|β|^2^ = 1. The operation of classical bits is different from qubits since they have a constant state between 0 and 1 at any given time. The core property of entanglement makes qubits sustain dependency in their states no matter the separation from each other. The capacity to transmit information via entanglement enables qubits to communicate at super speeds that result in quantum algorithms superior to classical computing abilities. The power of quantum states to enhance the chances of accurate solutions and eliminate wrong ones makes quantum algorithms very powerful, which is yet another reason why quantum interference is crucial in QC. In order to handle qubits in a manner that classical logic gates are unable to, QC uses quantum gates and circuits. The superposition is created by Hadamard gate, the CNOT gate establishes entanglement, and various Pauli gates, which rotate qubits around different axes. These gates form the foundation of quantum algorithms that solve complex problems much faster than classical computers. Due to its unique computational power, QC has promising applications in various fields. In the field of cryptography, conventional encryption techniques face competition from quantum algorithms like Shor’s algorithm, which can factor enormous numbers effectively. Quantum computing has the ability to speed up optimization procedures, boost data classification jobs, and increase pattern recognition in machine learning and artificial intelligence (AI).

In applications including autonomous systems, natural language processing, and image identification, DL, a subfield of artificial intelligence, has achieved impressive results. However, especially when working with large datasets, DLMs can demand a substantial amount of processing power for both training and inference. This is the point at which QC can offer substantial benefits. Quantum-enhanced DLMs integrate quantum feature extraction with classical neural networks to improve learning efficiency and generalization. In such hybrid models, a classical neural network extracts initial features from the input data, reducing the dimensionality and complexity before passing the processed information to a quantum layer. The quantum layer, designed using quantum circuits, further refines the feature extraction process by encoding the data into qubits and applying quantum transformations. These quantum transformations enable efficient representation learning, enhance the model’s capacity to identify intricate patterns in the data.

**Fig. 2:**
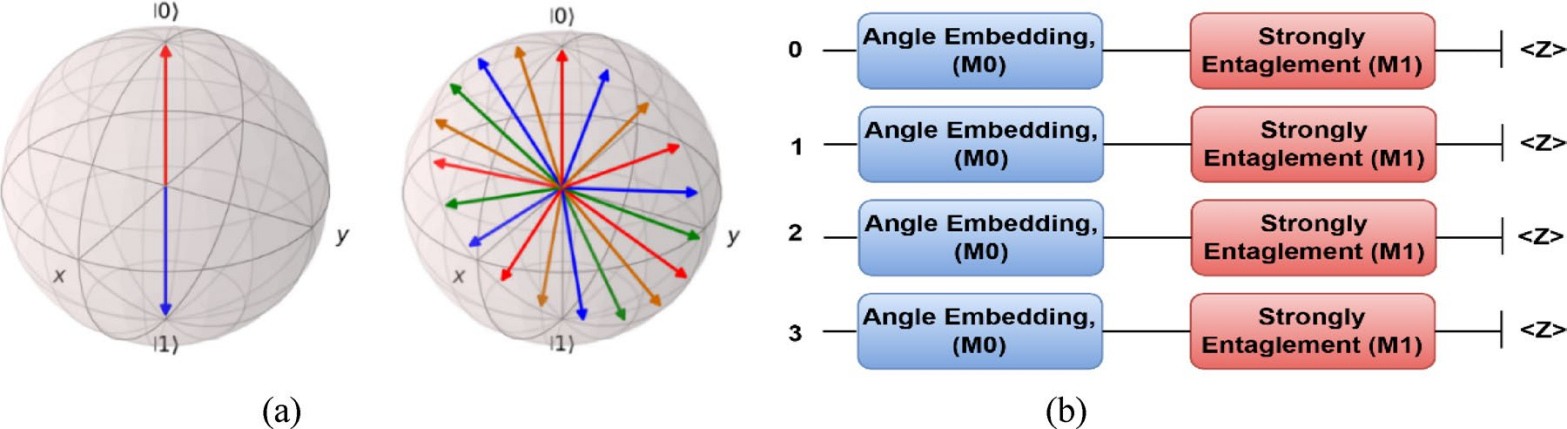
Bloch representation (**a**), and Quantum Circuit with Pauli Measurement (**b**).

Figure 2a shows two Bloch spheres, which are visual representations of qubit states. Here’s a breakdown of the key elements: Left Bloch Sphere, it represents a single qubit state. The blue arrow points downward (toward ∣1⟩), and the red arrow points upward (toward ∣0⟩). This suggests that the qubit state is a superposition or possibly an eigenstate of σ_z_ (Pauli-Z operator). The axes (x, y, z) are labelled, with the north pole being ∣0⟩ and the south pole being ∣1⟩. Right Bloch Sphere, it displays multiple qubit states as Bloch vectors. Each vector has a different color (blue, red, green, orange), indicating different qubit states. These vectors suggest various superpositions and phases of qubit states in the Bloch sphere representation. The qubit states are spread across the sphere, meaning different phase and amplitude variations. The Bloch sphere is a common way to visualize quantum states in a 2D space. Single-qubit states are points on or inside the sphere, while pure states are vectors on the sphere’s surface. The right image shows a set of qubits in different states, possibly from a quantum computation or entanglement visualization.

And Fig. 2b, shows the employed quantum circuit with Pauli measurement.

**Fig. 3:**
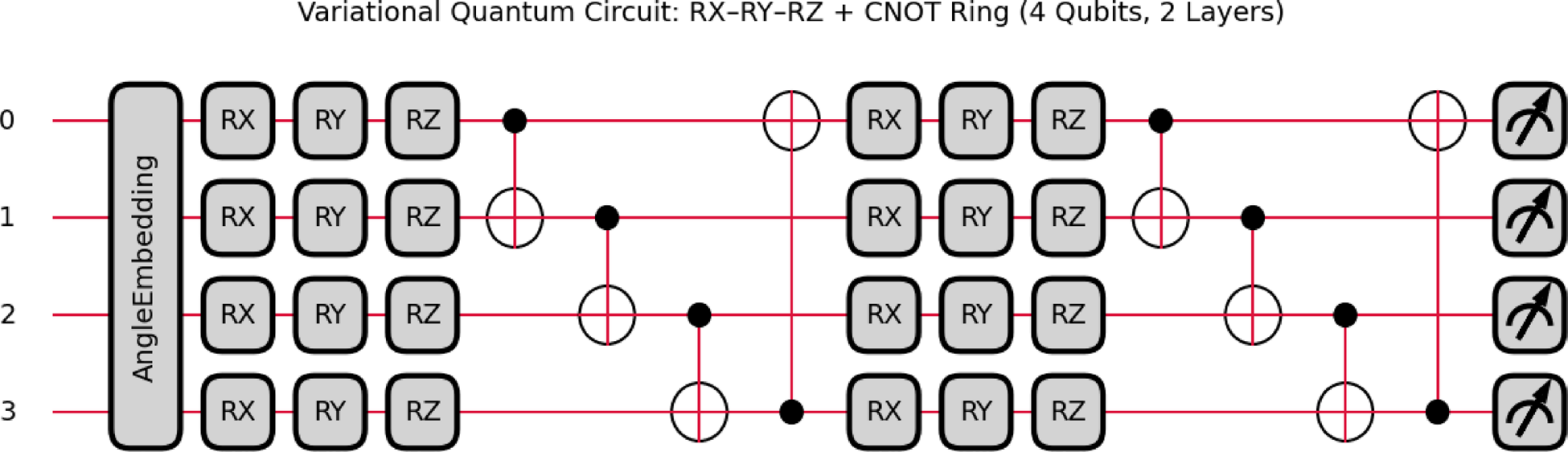
Structure of proposed Variational Quantum Circuit with 2 strongly entangling layer.

In the proposed VQEDTL framework, the quantum layer construction as in Fig. 3 includes the following steps,

**Algorithm 1:**
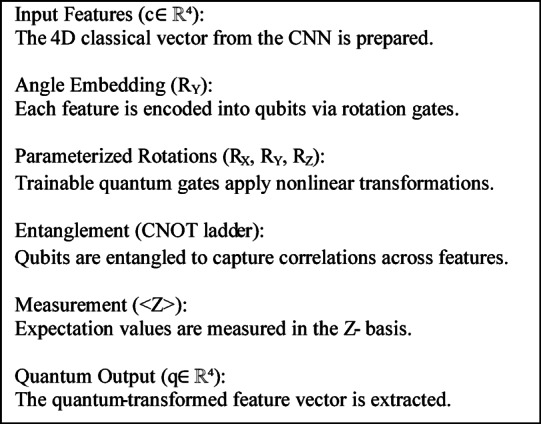
Workflow of gate operation in of Proposed Quantum Layer

#### Angle embedding M_n_ for n qubits

For an input sample with *n* features, the classical data vector is defined as,

1 $${M}_{n}=\left[{m}_{1}.{m}_{2},\dots ,{m}_{n}\right], {M}_{n}\in {R}_{n}.$$

Each element m_i_ is mapped to a qubit using an angle embedding operation, where a rotation around the Pauli-Y axis is applied:

2 $${R}_{y}{(m}_{i})=\text{exp}(-i\frac{{m}_{i}}{2}Y)$$

This transforms the initial qubit state ∣0⟩ into,

3 $$\mid {\psi }_{i}\rangle ={R}_{y}({m}_{i})\mid 0\rangle .$$

The joint quantum state of n qubits after embedding is,

4 $$\left| {\Psi_{embed} = \otimes_{i = 1}^{n} R_{y} \left( {m_{i} } \right)} \right|\left. 0 \right\rangle .$$

The quantum state after embedding for n qubits is,

5 $${R}_{y}{(\theta }_{i})=\left[\begin{array}{cc}\text{cos}(\frac{{\theta }_{i}}{2})& -\text{sin}(\frac{{\theta }_{i}}{2})\\ \text{sin}(\frac{{\theta }_{i}}{2})& \text{cos}(\frac{{\theta }_{i}}{2})\end{array}\right]$$

here, ∣0⟩| is the initial state of the qubits.

#### Parametrized Single-Qubit rotations

After embedding, each qubit undergoes trainable single-qubit rotations:

6 $${R}_{X}({\theta }_{i}^{x})=\text{exp}\left(-i\frac{{\theta }_{i}^{x}}{2}X\right)$$

7 $${R}_{Y}{(\theta }_{i}^{y})=\text{exp}\left(-i\frac{{\theta }_{i}^{y}}{2}Y\right)$$

8 $${R}_{z}{(\theta }_{i}^{z})=\text{exp}(-i\frac{{\theta }_{i}^{z}}{2}Z)$$

where, $\theta i=\{{\theta }_{i}^{x},{\theta }_{i}^{y},{\theta }_{i}^{z}\}$ are variational parameters optimized during training.

The collective local unitary applied across n qubits is:

9 $$U_{rot} \left( \Theta \right) = \otimes_{i = 1}^{n} R_{x} (\theta_{i}^{x} )R_{y} (\theta_{i}^{y} )R_{z} (\theta_{i}^{z} )$$

#### Strongly entangling layer

To induce correlations between features, a strongly entangling layer (SEL) is applied. This consists of a ring of CNOT gates interleaved with parametrized single-qubit rotations,

10 $${U}_{entangle}(\Theta )=\prod_{(i,j)\in \varepsilon }CNO{T}_{i,j}\cdot {U}_{rot}(\Theta )$$

where $\varepsilon$ defines the entangling topology (e.g., nearest-neighbor ring).

#### Layer stacking

To induce correlations between features, a strongly entangling layer (SEL) is applied. This consists of a ladder of CNOT gates interleaved with parametrized single-qubit rotations.

11 $$SEL(\Theta )={U}_{entangle}\boldsymbol{}(\Theta )\cdot {U}_{rot\boldsymbol{}}(\Theta ).$$

For L layers, the full quantum feature map unitary is,

12 $$U\left( {\Theta ,Mn} \right) = \mathop \prod \limits_{l = 1}^{L} SEL\left( \Theta \right) \cdot \otimes_{i = 1}^{n} R_{y} \left( {m_{i} } \right).$$

#### Final quantum state and measurement

The final quantum state is,

13 $$\left| {\left. {\Psi final} \right\rangle = U\left( {\Theta ,Mn} \right){\mid }} \right|\left. 0 \right\rangle^{ \otimes n} .$$

Measurements are performed in the computational (Pauli-Z) basis. The probability of outcomes is,

14 $$P\left( 0 \right) = \left| {0\Psi_{final} } \right|^{2} ,P\left( 1 \right) = \left| {1\Psi_{final} } \right|^{2} .$$

Expectation values across all qubits yield the quantum feature vector.

15 $${q}_{j}=\langle {\Psi }_{final}\mid {Z}_{j}\mid {\Psi }_{final}\rangle ,j=1,\dots ,n,q\in {\left[-\text{1,1}\right]}^{n}.$$

### VQEDTL design framework

**Fig. 4:**
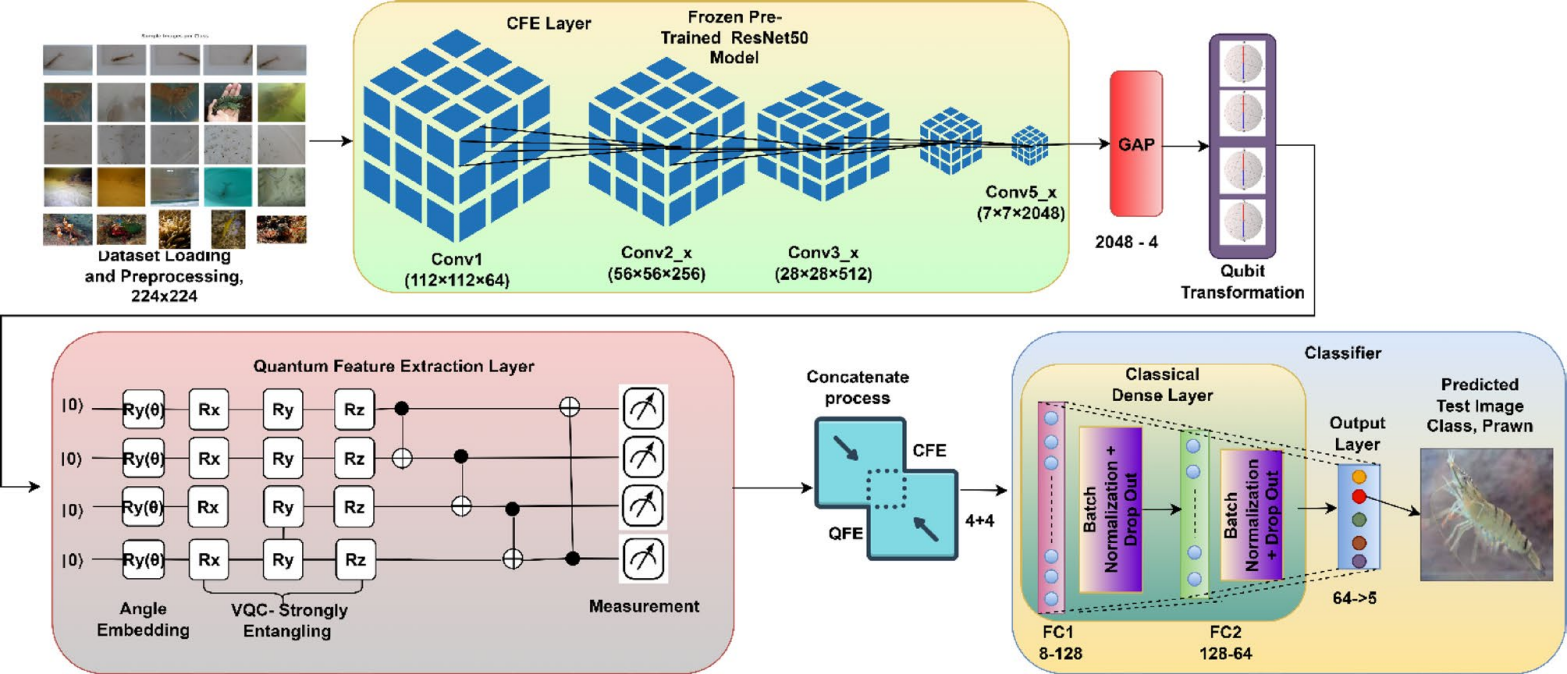
Architecture of proposed VQEDTL model for underwater small species classification.

The proposed VQEDTL model, Fig. 4 illustrates the architecture and work flow of proposed methodology. The model VQResNet50_10 emphasis the backbone of pretrained model, ResNet50, which utilizes the parameters of only 6 layers from the backbone and quantum circuit with 4 layers and so the total trainable layer here is 10. The proposed model consists of multiple layers, starting with input layer that takes image from pre-processed dataset of size 224 × 224 × 3. It passes through classical Resnet50 backbone that extracts features from the input images. The total parameter of this backbone is 23,859,333 and all of it are frozen, only used for feature extraction. This extracted feature undergoes global average pooling, GAP that reduces spatial dimension to a 2048-dimensional feature vector. A fully connected dense layer with 2048 feature vector maps into 4-dimensional output for quantum transformation requiring 8196 parameter (8192 weights and 4 biases). Following this, the output passes through a Quantum layer, Fig. 4 (keras_layer) and outputs 4-dimensional feature vector with 24 parameters which uses 2 entanglement layer (4 × 2 × 3). The outputs CFE layer and QFE layer are then concatenated, resulting in an 8-dimensional vector, (4 + 4). In the classifier, a second fully connected dense layer maps the 8-dimensional vector to 128 neurons, requiring 1152 parameters (1024 weights and 128 biases). A batch normalization layer follows, adding 256 additional parameters to normalize the activations. A dropout layer is applied for regularization, though it does not introduce any trainable parameters. The processed data is then passed through another dense layer, reducing the 128-dimensional representation to 64 neurons, requiring 8256 parameters (8192 weights and 64 biases). Another batch normalization layer follows, adding 128 parameters. A second dropout layer is applied for further regularization. Finally, the model outputs predictions through a final dense layer, mapping 64 features to 5 output classes, which involves 325 parameters (320 weights and 5 biases). The total number of trainable parameters in the model sums up to 18,337, primarily coming from the dense layers and batch normalization layers. The non-trainable parameters (23,588,096) are mainly from the ResNet50 backbone. This setup ensures that the model leverages ResNet50’s powerful feature extraction while keeping the trainable parameters minimal, making it efficient for training, especially on resource-constrained devices. The total parameter (TP) of CNN layer depends on kernel width and height, number of input and output channel and bias w.r.t output channel which is given by,

16 $${CNN}_{TP}=({K}_{H}\times {K}_{W}\times {C}_{in}\times {C}_{out})+b$$

For Dense, Fully Connected Layer, the total parameter is estimated by,

17 $${FC}_{TP}={n}_{i}* {n}_{o}+b$$

where, *n*_*i*_ is the input neuron, *n*_*o*_ is the output neuron and b, corresponding bias. As the batch normalization utilizes two learnable parameters, scaling and shifting for each feature, the total parameter obtained from batch normalization is given by,

18 $${BN}_{TP}=2*number of feature vector(F)$$

The GAP layer, drop out and concatenate layer do not contribute any parameter as it doesn’t introduce any training parameter and concatenation only fuses feature from CFE and QFE layer. As the ResNet backbone is frozen, non- trainable, the feature extraction happens efficiently without gradient updates, reduces the computational cost. The third dense layer has only 8256 parameters and this is less than the traditional classifier. The fused quantum layer, enhances the feature extraction, reduces the classical compute requirements. With this small trainable parameter, frozen backbone, q layer fusion for enhanced feature extraction and efficient dense layer, the proposed VQEDTL, VQResNet50_10 model achieves a light weight model for edge computing application. With that of existing work, the proposed work is the first hybrid quantum classical model with less trainable parameter and also applied to classify small underwater rare species, prawn and shrimp that supports aquaculture.

**Algorithm 2:**
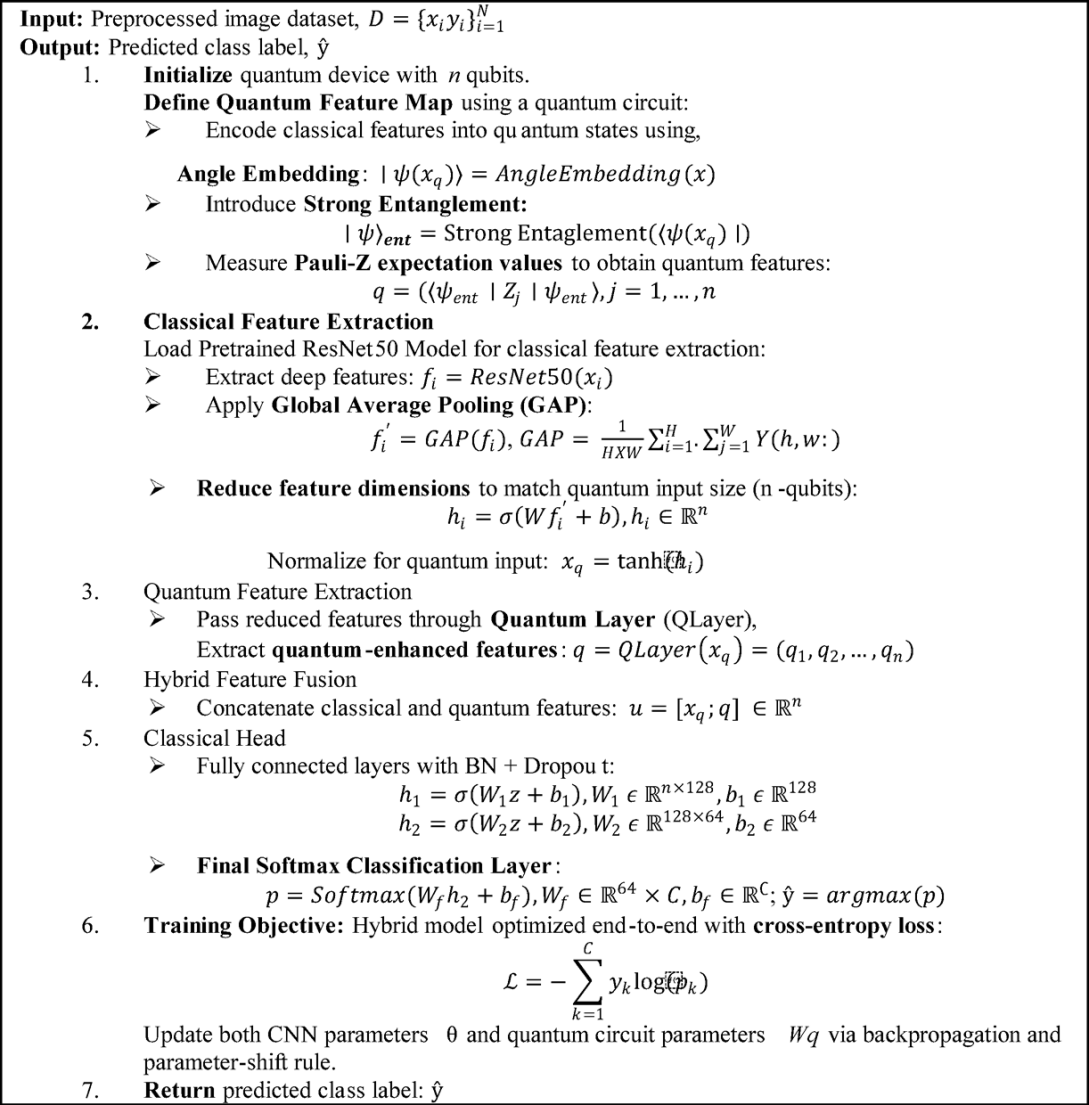
VQEDTL model for small underwater aquaculture species classification

### VQEDTL feature evolution and quantum—classical fusion

**Fig. 5:**
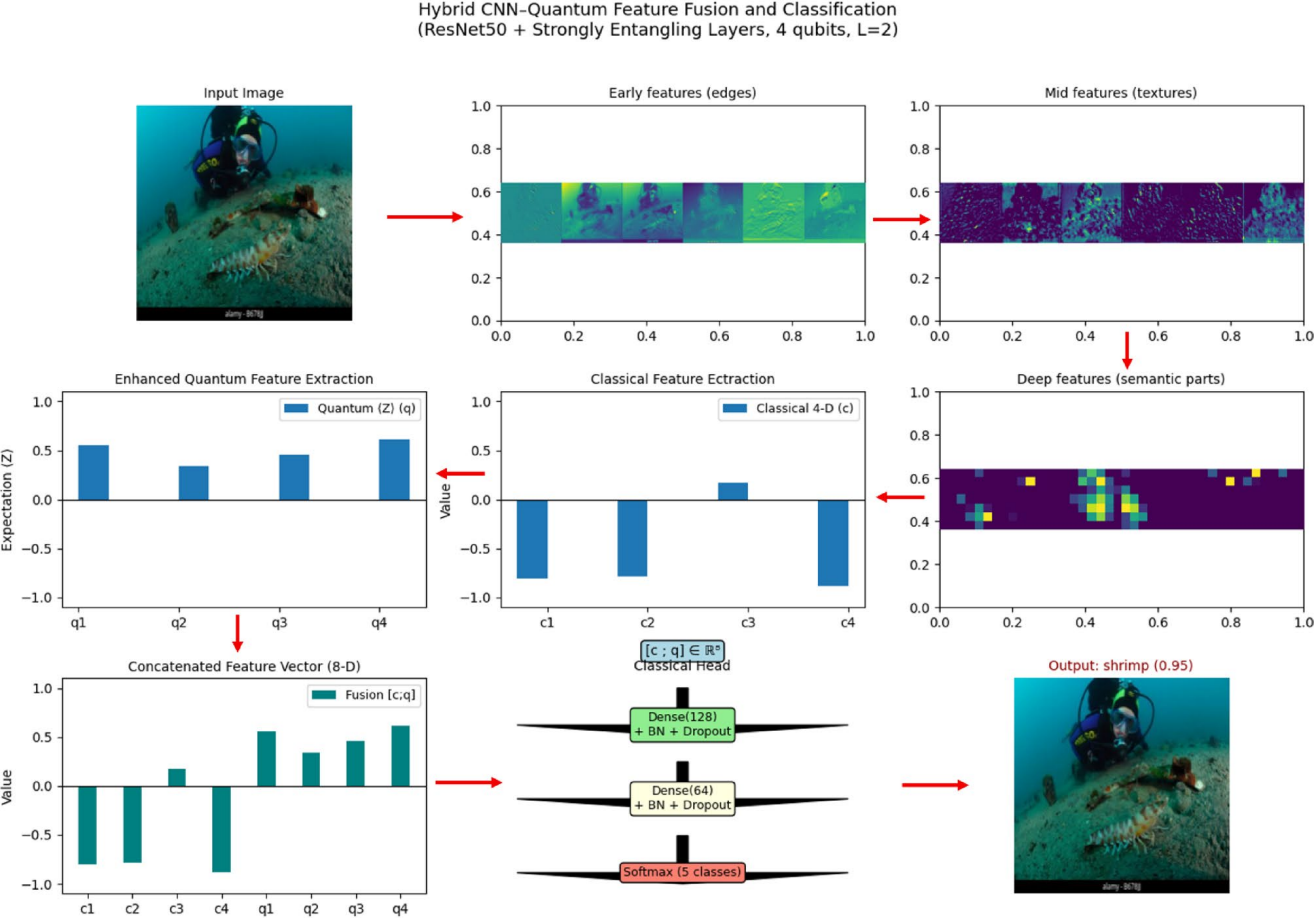
Feature evolution of proposed VQEDTL model for underwater small species classification.

To better illustrate the contribution of the proposed hybrid design, Fig. 5 presents a step-by-step visualization of the feature evolution from the input image through the CNN backbone, the classical projection, the quantum transformation, and finally the fusion stage. Let I, be the input image, $I\in {R}^{224\times 224\times 3}$ represents an underwater scene with low visibility and turbidity. Objects of interest (shrimps and prawns) are faint and partially occluded, making classification a challenging low-contrast problem.

#### CNN feature hierarchy

The pretrained model backbone extracts progressively abstract representations. Early layers respond to edges and contours, though underwater turbidity introduces background noise. Mid-level layers refine these into textural responses, beginning to separate object-like shrimp regions from water background. Deep layers activate on localized semantic parts (e.g., shrimp heads or body clusters), showing robustness to noise by suppressing irrelevant regions. For a given image III, the convolutional feature extractor produces hierarchical feature maps:

19 $${F}^{l}=ResNet5{0}_{l}^{\left(I\right)} ,l\in \{early,mid,deep\}.$$

where, Early features (*F*^*(early)*^): edge-like filters (7 × 7 convolutions), Mid features (*F*^*(mid)*^): texture and repeated pattern activations and Deep features (*F*^*(deep)*^): high-level semantic activations focusing on discriminative parts of the shrimp.

#### Classical projection

The deep CNN features are condensed via global average pooling and mapped through a dense layer with *tanh* non -linearity activation into a compact 4-dimensional vector c, to align with the quantum subsystem This low-dimensional representation encodes both positive and negative activations, reflecting alignment or suppression of semantic cues, and provides a stable input for quantum encoding.

20 $$z=GAP({F}^{(deep)})\in {R}^{d},d=2048.$$

21 $$c=tanh(Wz+b),c\in {R}^{4},$$

where $W\in {R}^{4\times d}, b\in {R}^{4}$. This gives the classical 4-D vector c = [c_1_, c_2_, c_3_, c_4_].

#### Quantum transformation

The classical vector c is embedded into a 4-qubit strongly entangling circuit. The output expectation values q ∈ [−1,1]^4^ represent a nonlinear entangled transformation of the features. In the illustrated case, negative entries in c are remapped into a smooth, all-positive distribution in q, confirming that the quantum layer introduces nontrivial fture space remapping and enhances expressivity through entanglement and superposition. Here Each c_i_​ is embedded into a single qubit by a rotation around the y-axis,

22 $${\text{Angle embedding}},\left| {\left. {\psi 0} \right\rangle = \otimes_{i = 1}^{4} R_{y} \left( {c_{i} } \right)} \right|\left. 0 \right\rangle .$$

where,

23 $${R}_{y}\left(\theta \right)=exp\left(-i\frac{\theta }{2}Y\right)=\left[\begin{array}{cc}\text{cos}\left(\frac{\theta }{2}\right)& -\text{sin}\left(\frac{\theta }{2}\right)\\ \text{sin}\left(\frac{\theta }{2}\right)& \text{cos}\left(\frac{\theta }{2}\right)\end{array}\right].$$

A strongly entangling layer applies a sequence of parameterized single-qubit rotations and CNOT entanglers,

24 $$U\left(\theta \right)=\prod_{l=1}^{L}\cdot \prod_{i=1}^{4}{R}_{z}\left({\theta }_{i,l}^{\left(1\right)}\right){R}_{y}\left({\theta }_{i,l}^{\left(2\right)}\right){R}_{z}\left({\theta }_{i,l}^{\left(3\right)}\right)\cdot CNOT-entangler.$$

Final Quantum state is given by,

25 $$\mid \psi (c,\theta )\rangle =U(\theta )\mid {\psi }_{0}(c)\rangle .$$

Each qubit is measured in the Pauli-Z basis,

26 $$q_{i} = \psi \left( {c,\theta } \right)Z_{i} \psi \left( {c,\theta } \right) \in \left[ { - 1,1} \right].$$

Output vector is, $q=({q}_{1}, {q}_{2}, {q}_{3},{q}_{4})\in {\left[-\text{1,1}\right]}^{4}$.

#### Fusion and classical head

The classical and quantum features are concatenated into an 8-dimensional hybrid vector u = [c; q]. This fused representation is then passed through two lightweight dense layers (128 and 64 neurons with batch normalization and dropout) before the softmax classifier. The fusion ensures that stable CNN features and quantum-enhanced encodings are jointly exploited for robust discrimination, particularly in underwater scenes. In feature mapping, Negative entries in ccc may be remapped to positive values in q. his happens because expectation values involve nonlinear trigonometric functions of the input c_i_​ and trainable parameters θ and thus, $q={f}_{quantum}(c;\theta )$ is a nonlinear entangled transformation enhancing model expressivity. The visualization confirms that the proposed VQEDTL pipeline is functioning as intended: CNN layers provide hierarchical abstraction, the classical projection compresses features into compact signals, the quantum circuit enriches these via nonlinear transformations, and the fusion mechanism delivers a balanced, expressive representation for classification. The experiment demonstrates that proposed VQEDTL models provide robust feature representations. Quantum outputs reshape classical features in a nontrivial manner, potentially offering improved class separability in noisy real-world domains such as underwater object recognition.

The presented Fig. 5 clearly illustrates how the proposed hybrid CNN–Quantum framework enhances feature extraction while reducing computational complexity. The baseline CNN (ResNet50) provides high-resolution semantic features, which are then passed through a quantum feature extraction module. The quantum layer introduces entanglement-based non-linear transformations that enrich the classical 4-D vector into a more expressive representation (⟨Z⟩ outputs). By fusing classical and quantum features into a compact 8-D vector, the model achieves a richer representation without significantly increasing dimensionality. This fusion allows the downstream classification head to operate on a lower-dimensional yet more discriminative feature space, effectively reducing computational cost compared to training large fully connected layers on high-dimensional CNN outputs. Overall, the figure demonstrates that the proposed VQEDTL model not only improves the representational power of the extracted features but also provides an efficient alternative to conventional deep heads, achieving higher accuracy with reduced complexity.

## Result and discussion

The proposed VQEDTL model was extensively evaluated using standard performance metrics to validate its classification capability and computational efficiency. Experimental results demonstrate the model’s effectiveness in accurately classifying underwater aquaculture shrimp and prawn images across multiple classes. Both qualitative and quantitative analyses are presented, highlighting not only the classification accuracy but also precision, recall, F1-score, and AUC values to ensure a balanced evaluation. Furthermore, computational measures such as FLOPs, inference time, throughput, and latency are analyzed to assess the practicality of the model for real-time deployment on resource-constrained platforms.

### Experimental setup

Training was conducted on an NVIDIA L4 GPU (24 GB VRAM) under CUDA 12.4 with cuDNN 9.1 acceleration. Batch normalization (momentum = 0.99, ϵ = 1 × 10^−3^) was applied after each dense block, coupled with a dropout rate of 0.5 to reduce overfitting. A mini-batch size of 32 was used, and training was capped at 10 epochs (119 iterations per epoch), with an early stopping criterion (patience = 5 epochs) applied to prevent unnecessary computation.

### Training and optimization

The VQEDTL model was trained in an end-to-end supervised learning framework, using categorical cross-entropy as the loss function and classification accuracy as the primary evaluation metric. All input images were resized to 224 × 224 and standardized through the ResNet50 preprocessing pipeline. To improve generalization, extensive data augmentation was applied, including random rotations (± 30°), width and height shifts (0.2), shear transformations (0.2), zoom variations (0.2), and horizontal flips (probability = 0.5), with nearest-neighbor interpolation used for edge filling.

Two optimization strategies were evaluated. The baseline used the Adam optimizer with β_1_ = 0.9, β_2_ = 0.999, ϵ = 1 × 10^−^⁷, and a fixed learning rate of 1 × 10^−3^. In addition, the AdaBoB optimizer was tested, designed to enhance stability by combining adaptive moment estimation with balanced weight decay. Its configuration included a learning rate of 1 × 10^−3^, β_1_ = 0.9, β_2_ = 0.999, ϵ = 1 × 10^−8^, γ = 1 × 10^−3^, and weight decay = 1 × 10^−4^. To stabilize optimization, an adaptive learning rate scheduler reduced the learning rate when the validation loss plateaued. Gradient clipping was employed to prevent exploding gradients, while layer normalization ensured controlled activation scaling across both classical and quantum layers.

Quantum circuits are inherently sensitive to decoherence, gate errors, and readout errors, which can degrade fidelity and impact hybrid model performance. In this work, noise was considered at two complementary levels. Circuit-level mitigation was addressed by employing shallow quantum circuits with only two strongly entangling layers and parameterized gates of limited depth, thereby reducing error accumulation and decoherence risk. Classical-domain regularization was addressed through dropout, which, while not directly eliminating quantum noise, prevents over-reliance on neurons that may capture spurious or noise-driven features from the quantum layer. Dropout enforces redundancy in feature learning, ensuring robustness to fluctuations in quantum outputs and acting as a statistical filter that reduces the propagation of noisy activations into the final decision boundary.

Hyperparameter tuning further refined convergence and generalization, Table 2. Learning rates of 1 × 10^−2^, 1 × 10^−3^, and 1 × 10^−4^ were explored, with 1 × 10^−3^ yielding the most stable performance. Batch sizes of 16, 32, and 64 were tested, with 32 selected as the optimal balance. The quantum circuit depth was set to two strongly entangling layers, though deeper circuits (≥ 3) may provide additional representational power at higher computational cost. Overall, these training and optimization strategies ensured robust convergence, stability, and reproducibility of the VQEDTL model, while balancing the trade-offs between performance and computational efficiency.

**Table 2:** Summary of hardware, software, and hyperparameter configurations used in the experimental setup.

Hardware environment	Software environment	Model hyperparameter	
GPU model: NVIDIA L4 (24 GB; 23034 MiB usable)	Python: 3.11 (system/venv)	Optimizer: Adam	Optimizer: AdaBoB
Driver/CUDA runtime: 550.54.15/CUDA 12.4	TensorFlow: 2.12.0/2.17.0 (pip)	Learning rate: 1.0 × 10^−3^	Learning rate: 1.0 × 10^−3^ (final_lr = 1.0 × 10^−3^)
cuDNN version: 9.1	Keras :2.12.0/3.5.0 (pip)	β_1_ = 0.9, β_2_ = 0.999, ε = 1e^−^⁷	β_1_ = 0.9, β_2_ = 0.999, ε = 1e^−8^
GPU temperature: 37 °C	NumPy: 1.23.5 (pip)	Batch size: 32	Batch size: 32
Power (usage/cap): 11 W/72 W	PennyLane: 0.40.0/0.42.3 (pip)	Max epochs: 10 (with early stopping)	Max epochs: 10 (with early stopping)
VRAM utilization: 0/23,034 MiB (idle)	PennyLane-Lightning: 0.40.0 (pip)	Dropout rate: 0.5 (dense layers)	Dropout rate: 0.5 (dense layers)
CPU/RAM: Intel Xeon (2 vCPUs, 53 GB RAM)	scikit-learn: 1.3.2 (pip)	Early stopping: patience = 5 epochs	Early stopping: patience = 5 epochs
Operating System: Ubuntu 20.04 (Colab VM)	Matplotlib: 3.7.3 (pip)	Weight init: Glorot uniform	Weight init: Glorot uniform
GPU core clock: ~ 735 MHz (Boost Clock)	Pandas: 2.0.3 (pip)	BatchNorm: momentum = 0.99, ε = 1e^−3^	BatchNorm: momentum = 0.99, ε = 1e^−3^
Memory clock/BW: 2250 MHz (9000 MHz effective)/300 GB/s		LR schedule: constant	γ = 1e^−3^, weight decay = 1e^−4^
CUDA cores: 7424			

### Performance metrics

To comprehensively evaluate the proposed VQEDTL model, multiple performance metrics are utilized. An overall measure of how well the model classifies images is estimated by metric Accuracy. Since accuracy by itself cannot provide precise model performance because of imbalanced datasets, other metrics like precision, recall, and F1-score are also evaluated. Recall assesses how many real examples of a class were properly identified, whereas precision examines how many of the projected instances of a class are truly correct. A more balanced measure is given by the F1-score, which is the balanced mean between precision and recall, particularly when there are differences between classes. The number of correctly and incorrectly classified instances for each class is represented by a confusion matrix to give a better insight into the performance of the model in classification. This enables the identification of the particular classes with respect to which the model performs poorly and this can be improved selectively. The detailed more assessment is achieved by each class’s ROC (Receiver Operating Characteristic) curve. The model’s ability to differentiate across classes is obtained by computing the AUC (Area Under the Curve) score. Better class separation is indicated by a higher AUC score, and the model’s overall performance in multi-class classification is assessed using the macro-averaged AUC. The number of arithmetic operation that a model utilizes to perform prediction is estimated by FLOPs, floating point operations. The computational complexity of the model is well analyzed from the estimated FLOP. For a more detailed assessment, each class’s ROC (Receiver Operating Characteristic) curve is also presented. The model’s ability to differentiate across classes is obtained by computing the AUC (Area Under the Curve) score. Better class separation is indicated by a higher AUC score, and the model’s overall performance in multi-class classification is assessed using the macro-averaged AUC. The number of arithmetic operation that a model utilizes to perform prediction is estimated by FLOPs, floating point operations. The computational complexity of the model is well analyzed from the estimated FLOP. The efficacy of the VQEDTL models can be thoroughly evaluated by combining various performance measures. The model is a well-generalized classifier, as evidenced by its high precision and recall across all classes. A thorough assessment framework is offered by these metrics taken together to guarantee the accuracy of the quantum–classical method for classifying underwater images.

27 $$Accuracy=\frac{TP+TN}{TP+FP+TN+FN}$$

28 $$Precision (P)=\frac{TP}{FP+TP}$$

29 $$Recall (R)=\frac{TP}{FN+TP}$$

30 $${F}_{1}-score=\left(1+{\beta }^{2}\right).P.\frac{R}{{\beta }^{2}}.\left(P+R\right)=\frac{2PR}{P+R}$$

$\beta$, is the weight factor that determines the importance of Recall Vs Precision.

31 $$FLOPs\approx 2\times (No. of Trainable Parameters)$$

In addition to the above classification and complexity measures, further evaluated the model efficiency and training dynamics of the proposed VQEDTL model to ensure its suitability for real-time deployment in resource-constrained environments.

Model Efficiency Metrics, the speed and latency of the model are critical in real-world underwater applications. To quantify this, the following metrics are computed, average inference time (t_avg_), Throught-put, and Latency (L).

32 $$\text{Average Inference Time }{\text{t}}_{\text{avg}}=\frac{1}{R}\sum_{r=1}^{R}{(t}_{1}^{r}-{t}_{0}^{r})$$

where ${t}_{0}^{r}$ and ${t}_{1}^{r}$ denote the start and end times of inference for the r-th repetition, and R is the number of repetitions.

33 $$Throughput, T=\frac{N}{{t}_{avg}}.$$

where, throughput is the number of images processed per second and N is the number of test image and latency, is the per image prediction time and given as,

34 $$Latency, L=\frac{{t}_{avg}}{N}\times 1000 \left(ms/image\right).$$

These metrics collectively indicate whether the model can operate at real-time speeds for underwater image classification.

Training dynamic Metrics, examine the model’s learning behavior and generalization, the following measures are introduced: Convergence speed (E_90_) is the epoch number when the validation accuracy first reaches 90% of its maximum value and is given by,

35 $$E_{90} = min\left\{ {eVal_{Acc} \left( e \right) \ge 0.9 \times \underbrace {max}_{e}Val_{Acc} \left( e \right)} \right\},$$

Stability (${\sigma }_{val}$) is the the standard deviation of validation accuracy over epochs, which reflects the robustness of the training process,

36 $${\sigma }_{val}=\sqrt{\frac{1}{M}\sum_{i=1}^{M}{\left(Va{{l}_{acc}}_{i}-Va{l}_{acc}\right)}^{2}}.$$

where M is the total number of epochs and overfitting tendency ($\Delta Acc$), id the gap between training and validation accuracy in the final epoch,

37 $$\Delta Acc=Ac{c}_{train}^{M}-Ac{c}_{val}^{M}.$$

A smaller *ΔAcc* indicates better generalization capability, while larger values suggest overfitting.

### VQEDTL model observation analysis

The performance of different Variational Quantum (VQ) models, VQResNet50_10, VQInceptionResNetV2_10, VQMobileNet_10, and VQDenseNet201_10 vary significantly based on hyperparameters and architectural complexity. Table 3 gives the insights of the proposed quantum fusion model for classification of small underwater species and the recently introduced ISONet^37^. ISONet introduces controllable input spatial over-parameterization in convolutional layers during training, which integrates momentum acceleration and adaptive learning rate mechanisms. Importantly, during inference, the over-parameterized structure is folded into a standard convolutional layer, enabling efficiency gains without increasing inference complexity.

**Table 3:** Comparison of various proposed VQEDTL models with ISONET.

Model hyper parameter	ISONET	VQResNet50_10	VQInceptionResnetv2_10	VQMobileNetv2_10	VQDenseNet201_10
Classical layer	17	6	6	6	6
Quantum layer	–	4	4	4	4
Qubit	–	4	4	4	4
Train parameter	3,278,371	18,337	16,289	15,265	17,825
Training time	40 min	74 min	130.5 m	46 min	123.5 min
Training loss	0.5505	**0.0546**	0.2793	0.0987	0.0813
Validation loss	0.5358	0.0437	0.2333	0.1548	0.0892
Test loss	0.5138	0.0247	0.2573	0.1413	0.0754
Train accuracy	0.9404	0.9837	0.8856	0.9710	0.9763
Valid accuracy	0.9468	**0.9908**	0.8890	0.9532	0.9752
Test accuracy	0.9460	0.9926	0.8827	0.9609	0.9814
Precision	0.94	0.99	0.87	0.96	0.98
Recall	0.93	0.99	0.88	0.96	0.98
F1score	0.93	0.99	0.87	0.96	0.98

Significant values are in bold.

#### Training and convergence analysis

ISONet achieves strong performance with reduced model size (3.27 M parameters) and relatively short training time (40 min), while maintaining competitive accuracy (94.6% test accuracy, 0.93 F1-score). This confirms its effectiveness as a state-of-the-art lightweight architecture. However, VQEDTL variants consistently outperform ISONet in key evaluation metrics. Here, VQResNet50_10 exhibits the fastest convergence, with a lowest training loss of 0.0546, indicating effective learning and minimal overfitting. VQDenseNet201_10 follows closely with 0.0813 training loss an accuracy of 97.63% indicating strong feature extraction. VQMobileNet_10, despite being lightweight, performs efficiently with 97.10% training accuracy. However, VQInceptionResNetV2_10 struggles with slower convergence, showing the highest training loss (0.4858) with lowest accuracy 88.56%, suggesting difficulties in optimization when integrated with quantum layers. Training time varies significantly, with VQMobileNetV2_10 requiring only 46 min, whereas VQInceptionResNetV2_10 takes the longest at 130.5 min, likely due to its deeper architecture and higher computational demand.

The results, Table 3, demonstrate that, the integration of variational quantum circuits in VQEDTL enhances the network’s representational capacity, allowing it to capture subtle morphological distinctions critical for small-species underwater classification. This advantage is evident in higher validation/test accuracies and superior F1-scores compared to both ISONet and conventional CNNs. Overall, these findings highlight that while ISONet establishes an efficient and competitive classical baseline, the proposed VQEDTL framework achieves state-of-the-art accuracy and robustness, thereby validating the unique advantage of quantum-enhanced designs in challenging underwater image classification tasks.

#### Precision, recall and F1-score

**Fig. 6:**
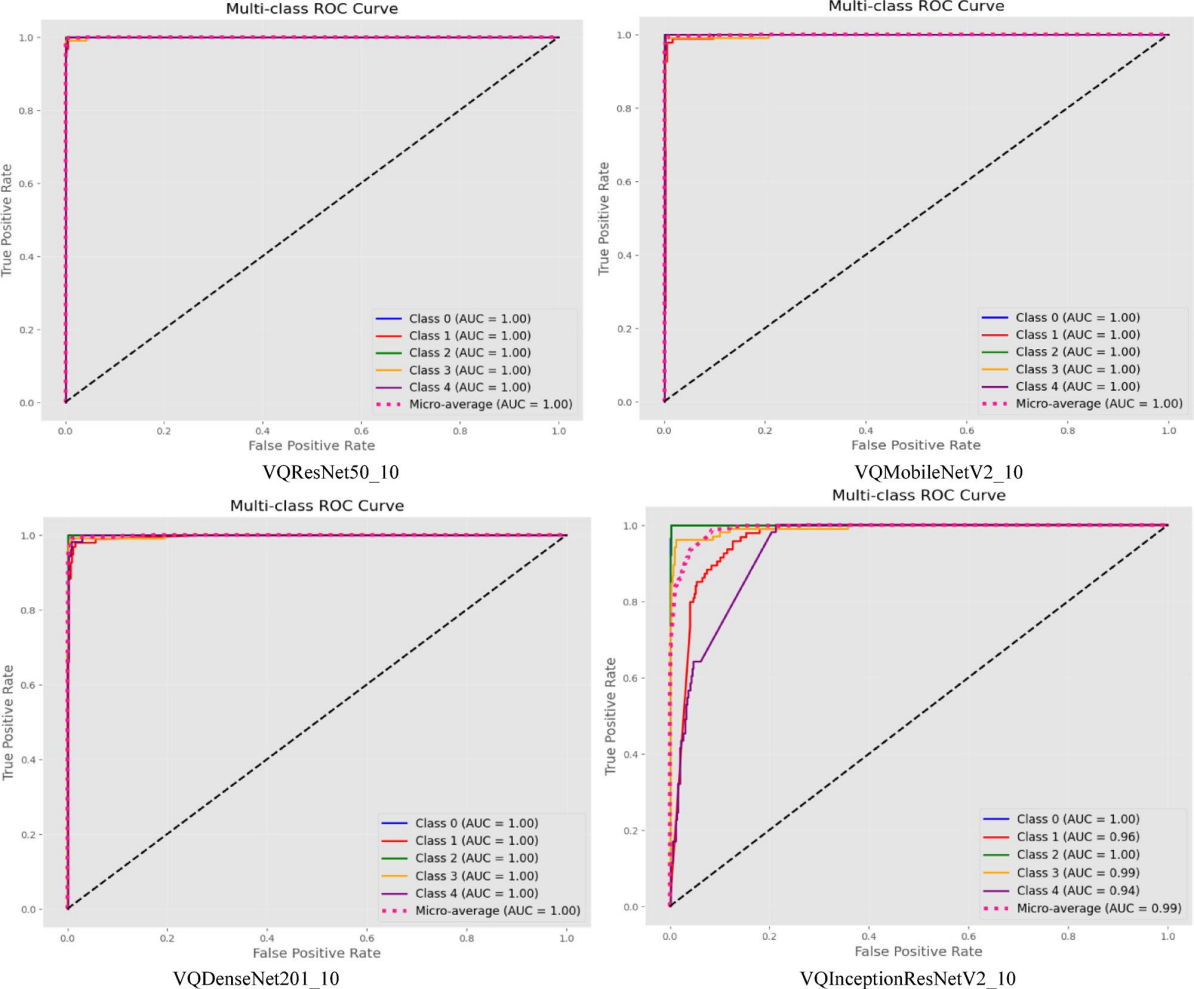
Visualization of ROC of Proposed VQEDTL Models.

**Fig. 7:**
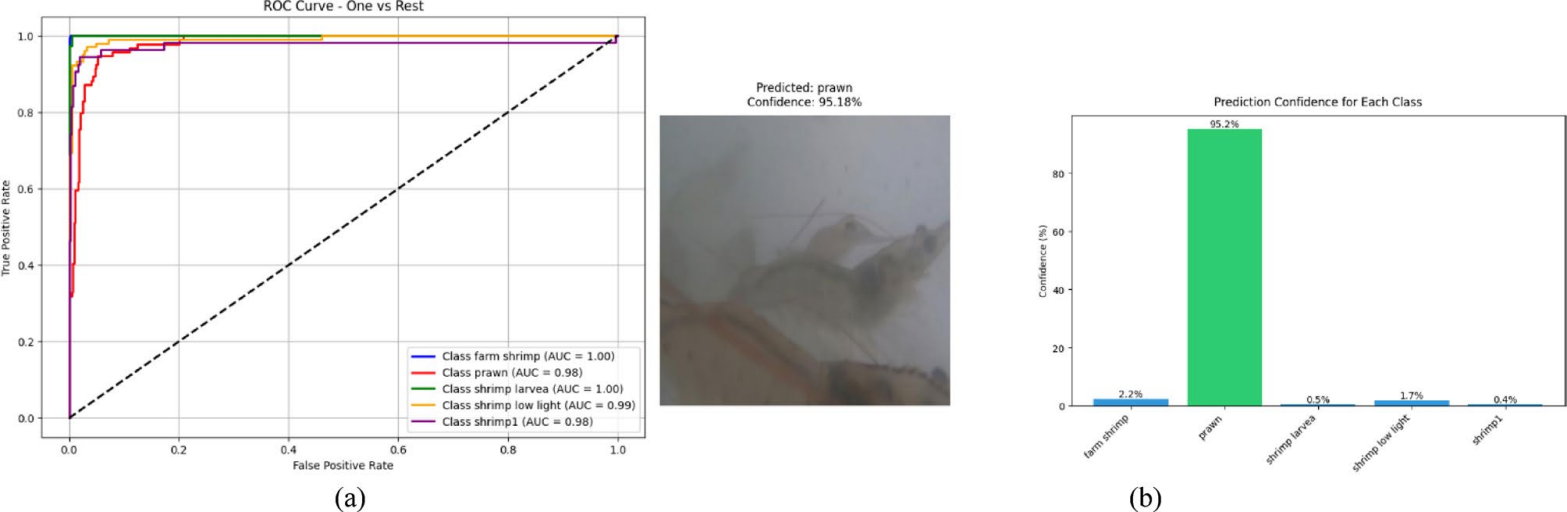
ROC curve (**a**) and Predicted Test image (**b**) by ISONet.

**Fig. 8:**
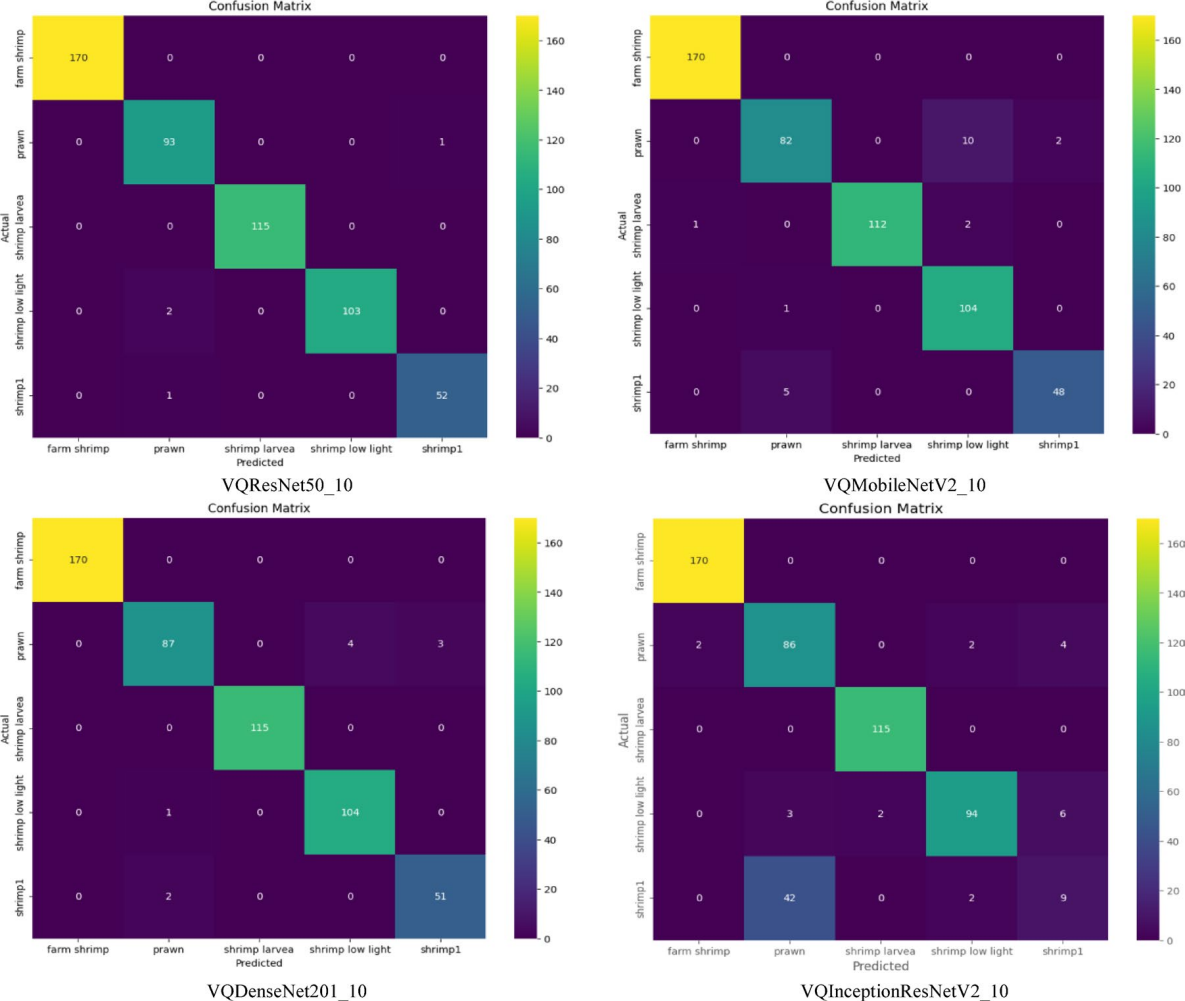
Confusion Matrix of Proposed VQEDTL Models.

**Fig. 9:**
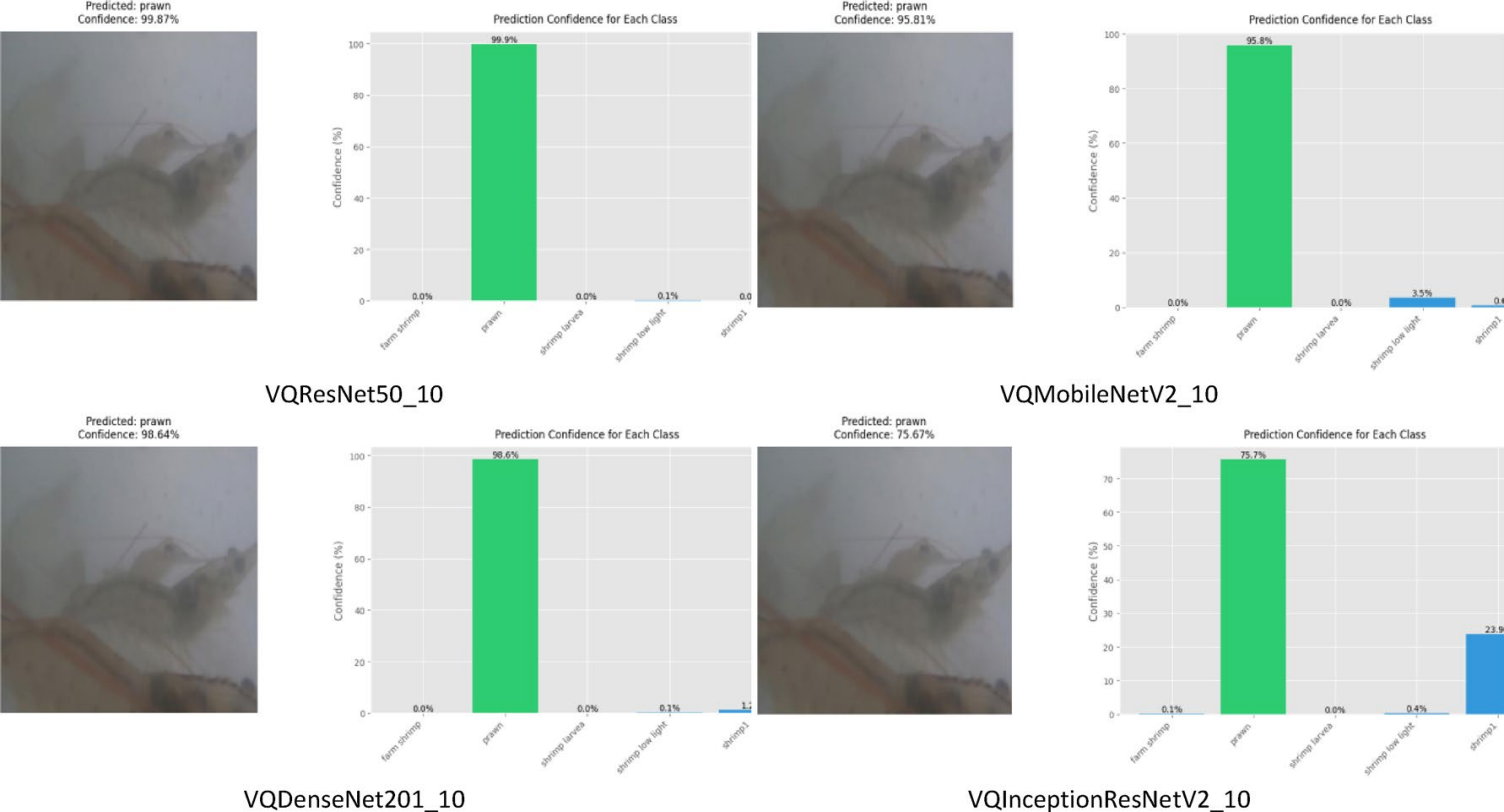
Predicted class of test images of different proposed VQEDTL Model.

VQResNet50_10 demonstrates the best overall precision, recall, and F1-score, Table 3, indicating high robustness across all classes. VQDenseNet201_10 follows closely with 0.98 in all metrics, showing that its deeper architecture supports quantum feature extraction well. VQMobileNetV2_10 also maintains strong results with 0.96 across all metrics, ensuring efficiency without significant performance trade-offs, where VQInceptionResNetV2_10 lags behind, scoring the lowest (0.87–0.88), further confirming its instability. Figure 6, presents ROC curve for proposed VQEDTL models, Fig. 7a, b shows the ROC curve and test image prediction of ISONET. In Fig. 8, each confusion matrix represents the classification performance of a proposed VQEDTL variants. The diagonal elements indicate the correctly classified samples and the off-diagonal elements showcase the misclassified samples. A strong diagonal means good performance, whereas high off-diagonal values indicate frequent misclassifications. Figure 9 displays the Predicted class of test images of different proposed VQEDTL Model. The figure implies that, VQResNet50_10 is the most confident model, VQInceptionResNetV2_10 has the most uncertainty, meaning it is less decisive. VQMobileNetV2_10 and VQDensNet201_10 show a balance between confidence and alternative class probabilities.

#### Generalization and overfitting trends

The results, Table 3 show distinct differences in generalization between the classical baseline (ISONET) and hybrid quantum–classical models. ISONet achieved strong generalization with balanced training and test accuracy (~ 94–95%), but at the expense of high parameter count (> 3.2M) and longer training time. VQResNet50_10 achieves the highest validation accuracy of 99.08%, though with a slightly higher validation loss of 0.0437, indicating strong learning. VQDenseNet201_10 maintains an impressive validation accuracy of 97.52% with the lowest validation loss (0.0892), suggesting excellent generalization capability. VQMobileNetV2_10, known for efficiency, achieves 95.32% validation accuracy, making it a viable model with a moderate validation loss of 0.1548. Conversely, VQInceptionResNetV2_10 lags with 88.90% validation accuracy, indicating suboptimal quantum integration

### Efficacy analysis of the proposed quantum structure

**Fig. 10:**
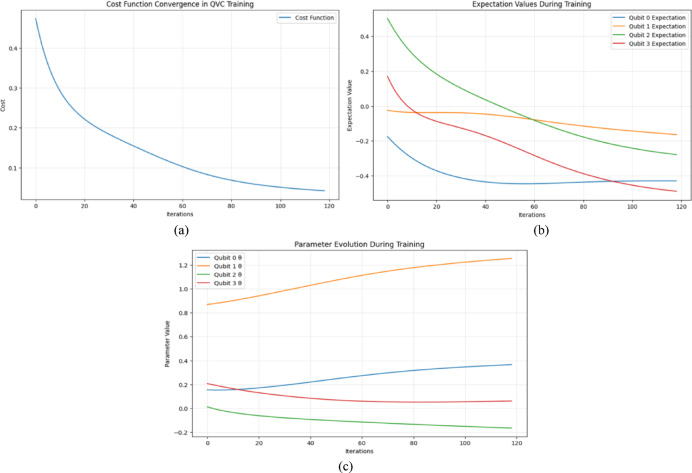
Efficacy Analysis of Proposed Quantum Structure, codt function convergence (**a**), expectation va;ue of individual bits (**b**) and parameter evolution during training (**c**).

The effectiveness and stability of the proposed Quantum Feature Extraction (QFE) layer are thoroughly demonstrated through the three subplots shown in Fig. 10, (i) the cost function convergence, (ii) the expectation values of individual qubits, and (iii) the parameter evolution during training. These plots provide a comprehensive view of how the quantum layer learns and adapts throughout the training process. The cost function convergence plot shows a smooth and gradual decrease in the cost over iterations. This indicates that the variational quantum deep transfer model, successfully minimizing the loss function, reflecting effective learning and optimization. The absence of sharp oscillations or flat regions suggests that the training does not suffer from barren plateaus, a common challenge in quantum variational circuits and thus confirming that the selected optimizer and learning rate are well-suited for the task. The expectation values plot offers insights into how each qubit behaves during training. The diverging trajectories of the expectation values across the four qubits reveal that different qubits are learning distinct aspects of the data. Some qubits show stronger fluctuations and convergence behavior, indicating their dominant contribution to encoding relevant features. This illustrates the distributed and parallel nature of quantum learning, where multiple qubits collaboratively contribute to feature extraction but may do so with varying intensities based on their entanglement and interaction patterns. The parameter evolution plot displays how the variational parameters (θ) of the quantum circuit evolve over time. The continuous and adaptive changes in parameter values further reinforce that the circuit is actively learning. The variation in the rate and direction of parameter adjustments suggests that certain qubits or layers are more critical for learning complex patterns, while others may serve supporting roles. This dynamic behavior confirms the flexibility and responsiveness of the QFE layer in adapting to the data’s underlying structure. Together, these plots validate the performance and reliability of the quantum layer within the hybrid model. The QFE layer not only learns meaningful quantum features but does so in a stable, interpretable, and efficient manner.

### Impact of quantum feature mapping on representation and robustness

**Fig. 11:**
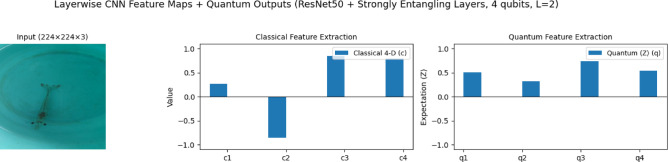
Visualization of Classical and Quantum Feature Extraction for Underwater Aqua Species Image Classification.

The comparison between classical and quantum feature spaces underscores the stabilizing role of the quantum layer in representation learning. The classical 4D vector (c1–c4) shows high variance, with one strongly negative activation, c2, one strongly positive c3, and moderate values for the remaining features. After quantum transformation, the outputs (q1–q4) are redistributed into a narrower, balanced range, which implies that the quantum layer smooths extreme fluctuations while preserving key discriminative signals. For example, the strong positive activation of, c3 is retained in, q3 whereas the strongly negative, c2 is reframed into a positive expectation value, q2. This mapping not only preserves informative features but also converts destructive activations into constructive signals, enhancing robustness against unstable decision boundaries. With only two entangling layers (L = 2), the outputs remain smooth and stable, confirming that shallow quantum circuits can capture correlations without over-amplification or noise accumulation. Figure 11 illustrates this transformation of ResNet50-derived classical features into quantum feature representations using a 4-qubit circuit. The results highlight two advantages of the proposed design: (i) essential discriminative information is retained, and (ii) extreme variations are moderated into homogenized quantum signals. Together, these properties demonstrate that the VQEDTL framework enhances feature representation while maintaining computational efficiency, making it well-suited for deployment on NISQ devices.

### Analysis of proposed model with baseline CNN models

#### Comparison of proposed VQEDTL, VQResNet50_10 models with baseline CNN models and ISONet

Table 4, presents a comparison between classical DLMs (ResNet50, InceptionResNetV2, MobileNet, and DenseNet201) with proposed VQResNet50_10, VQEDTL model which integrate quantum layers for enhanced extraction. The key parameters analyses include training parameters, FLOPs, inference time, training time and validation accuracy. The proposed VQEDTL framework achieve lightweight design without sacrificing performance. As shown in Table 4, the VQResNet50_10 model has only 18,337 trainable parameters and 36,674 FLOPs, representing a > 99% reduction compared to conventional CNNs such as ResNet50 (23.8 M parameters; 47.6 M FLOPs) and InceptionResNetV2 (54.5 M parameters; 108.9 M FLOPs). The inference time of 0.295 s is significantly faster than both ResNet50 (1.19 s) and ISONet (2.609 s). Although ISONet is specifically designed as a lightweight model (3.2 M parameters; 65.6 k FLOPs), our hybrid VQEDTL achieves an even lower computational footprint while improving accuracy.

**Table 4:** Comparison of VQRESNET50_10 model with classical DLMs.

Model	Training parameter	FLOPs	Inference time (s)	Validation accuracy (%)
ResNet50	23,805,829	47,611,658	1.19	95.6
MobileNetV2	24,31,301	4,862,602	0.95	85.69
InceptionResNetV2	54,481,893	108,963,786	3.499	84.77
DenseNet201	18,34,781	366,955,62	3.58	92.48
ISONet	3,278,371	65, 56,742	2.609	94.40
VQ ResNet50_10	18,337	36,674	0.295	99.08

#### Accuracy and feature representation

Despite its compact architecture, VQResNet50_10 achieves the highest validation accuracy of 99.08%, outperforming all classical baselines including ResNet50 (95.6%), DenseNet201 (92.48%), InceptionResNetV2 (84.77%), MobileNetV2 (85.69%), and ISONet (94.40%), Tables 4 and 5. This highlights the strength of the quantum–classical fusion layer, which enhances feature representation by capturing complex correlations between features that are not well modeled in purely classical CNNs. Comparative analysis of classical vs. quantum feature maps (Fig. 11) illustrates that quantum embedding produces richer and more balanced features, supporting the claim that VQEDTL improves representation quality.

**Table 5:** Comparison of VQEDTL Variants with baseline DLMs.

Metric	ISONet	Classical ResNet50	VQ ResNet50_10	Classical MobileNetV2	VQ MobileNetV2_10	Classical Inception ResNetV2	VQ Inception ResNetV2_10	Classical DenseNet201	VQ DenseNet201_10
Total params	3,278,371	23,859,333	**23,606,433**	2,431,301	**2,273,633**	54,542,821	**54,353,409**	18,577,221	**18,340,193**
Training params	3,278,371	23,805,829	**18,337**	2,396,805	**15,265**	54,481,893	**16,289**	18,347,781	**17,825**
FLOPs	65, 56,742	47,718,66	**36,674**	48,62,602	**30,530**	10,89,63,786	**32,578**	3,66,95,562	**35,650**
Inference time (s)	2.09	1.19	**0.295**	0.95	**0.259**	3.499	**0.655**	3.58	**0.589**
Validation Acc(%)	94.7	95.6	**99.08**	85.69	**95.32**	84.77	**88.9**	92.48	**97.52**

Significant values are in bold.

#### Robustness to underwater distortions

**Fig. 12:**
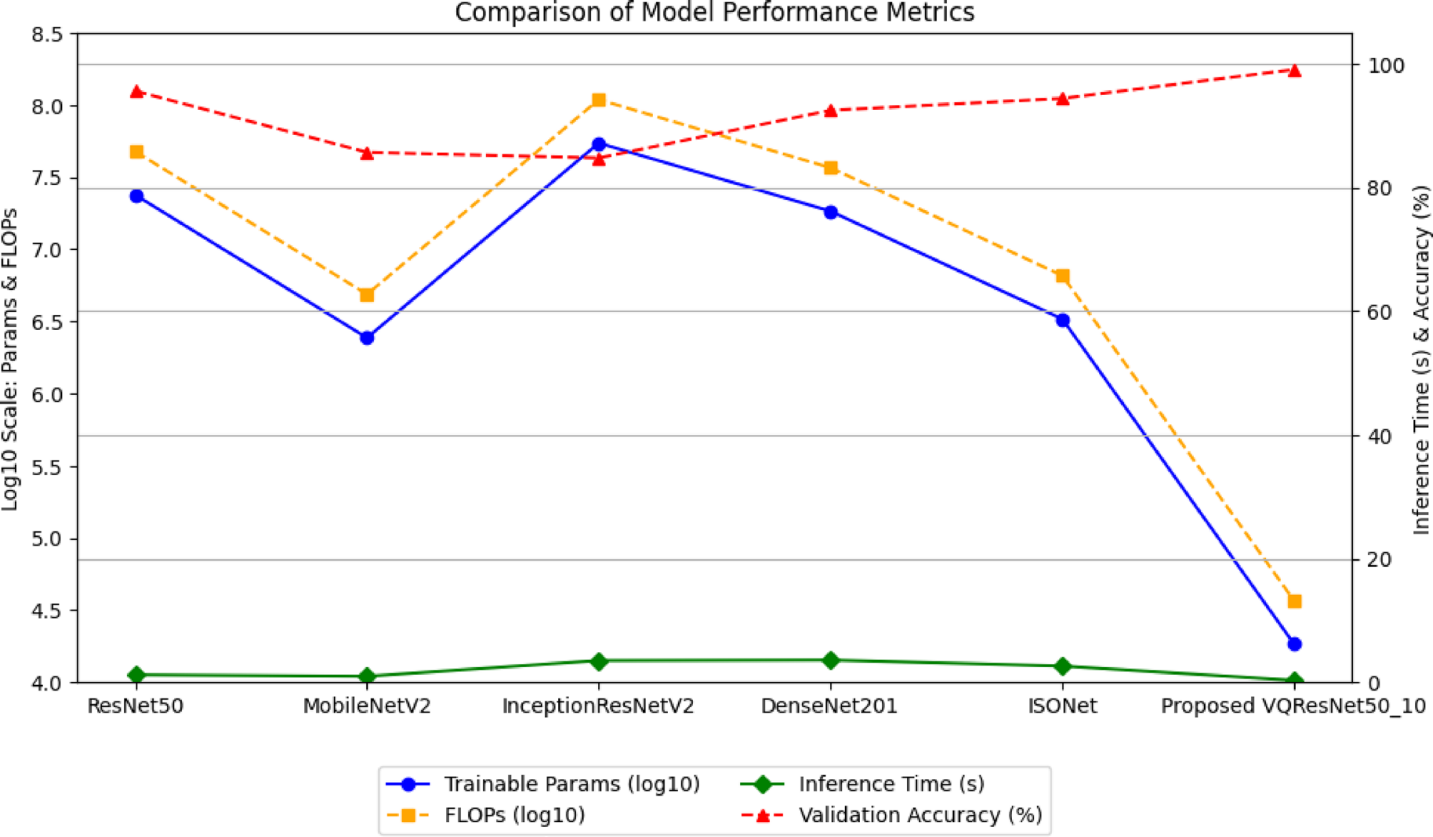
Comparison of Performance Metrics of Proposed VQResNet50_10 Model with Classical DLMs.

**Fig. 13:**
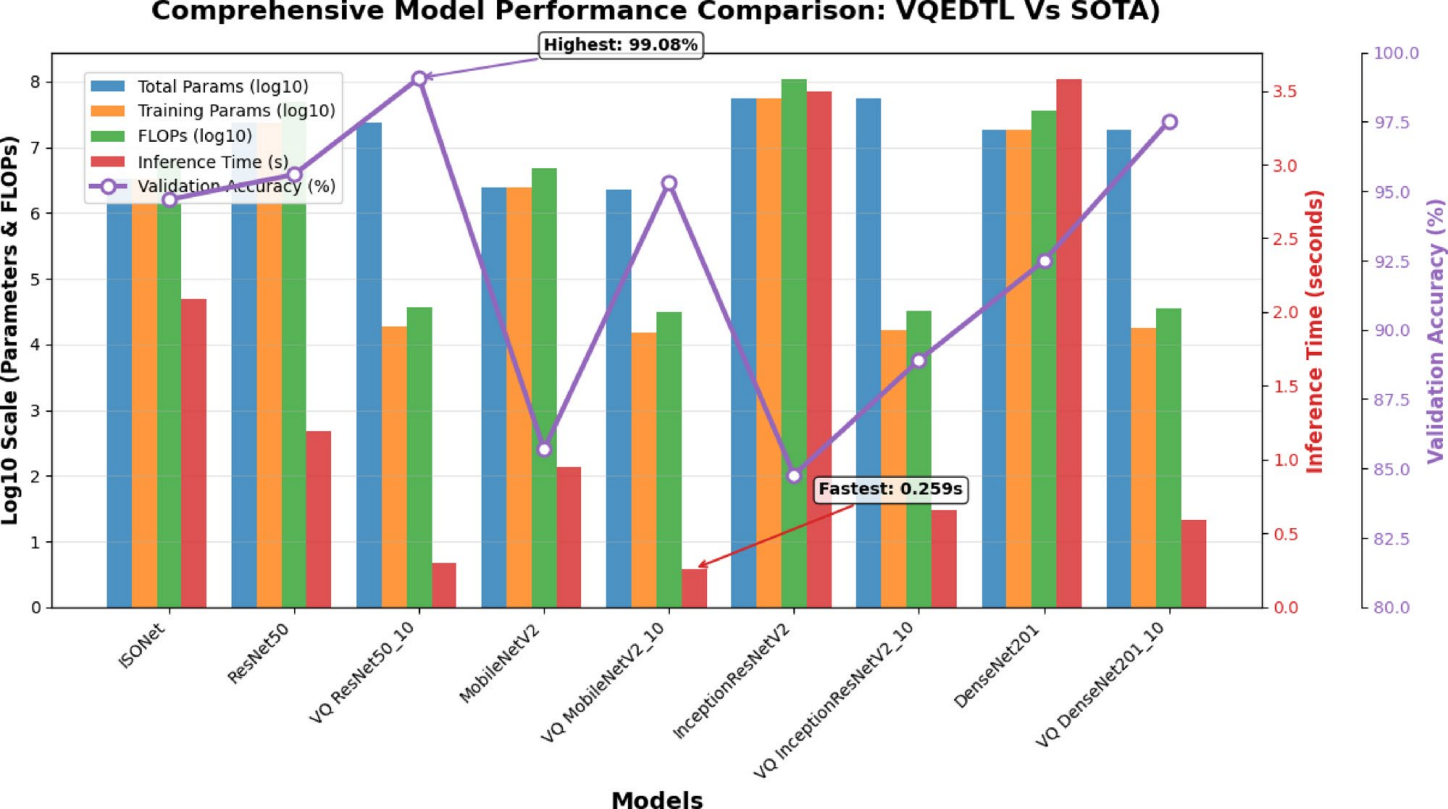
Computational Efficiency of Proposed VQEDTL Variance with SOTA Models.

Underwater images suffer from low visibility, scattering, and illumination variability. VQEDTL demonstrates strong resilience in such conditions, as shown by ROC curve analysis, Fig. 6 and confusion matrices, Fig. 8, where the model consistently achieves AUC scores above 0.97 across all classes. Minority classes that are more affected by noise (e.g., shrimp larvae, low-light shrimp) showed notably improved recognition with VQEDTL compared to ISONet and MobileNetV2 (Table 4). This confirms that quantum-enhanced feature extraction strengthens robustness against underwater distortions. Table 5, presents the overall model analysis across classical and variational quantum (VQ) architectures. The findings show that VQ variants continuously maintain or increase validation accuracy while achieving significant decreases in trainable parameters and FLOPs as well as noticeably faster inference times. This demonstrates the suggested models’ effectiveness, compactness, and scalability, which make them especially appropriate for settings with limited resources. Figures 12 and 13, showcase the proposed VQEDTL variant efficiency over other SOTA models.

#### Weight distribution analysis of proposed lightweight VQEDTL model, VQResNet_10

**Fig. 14:**
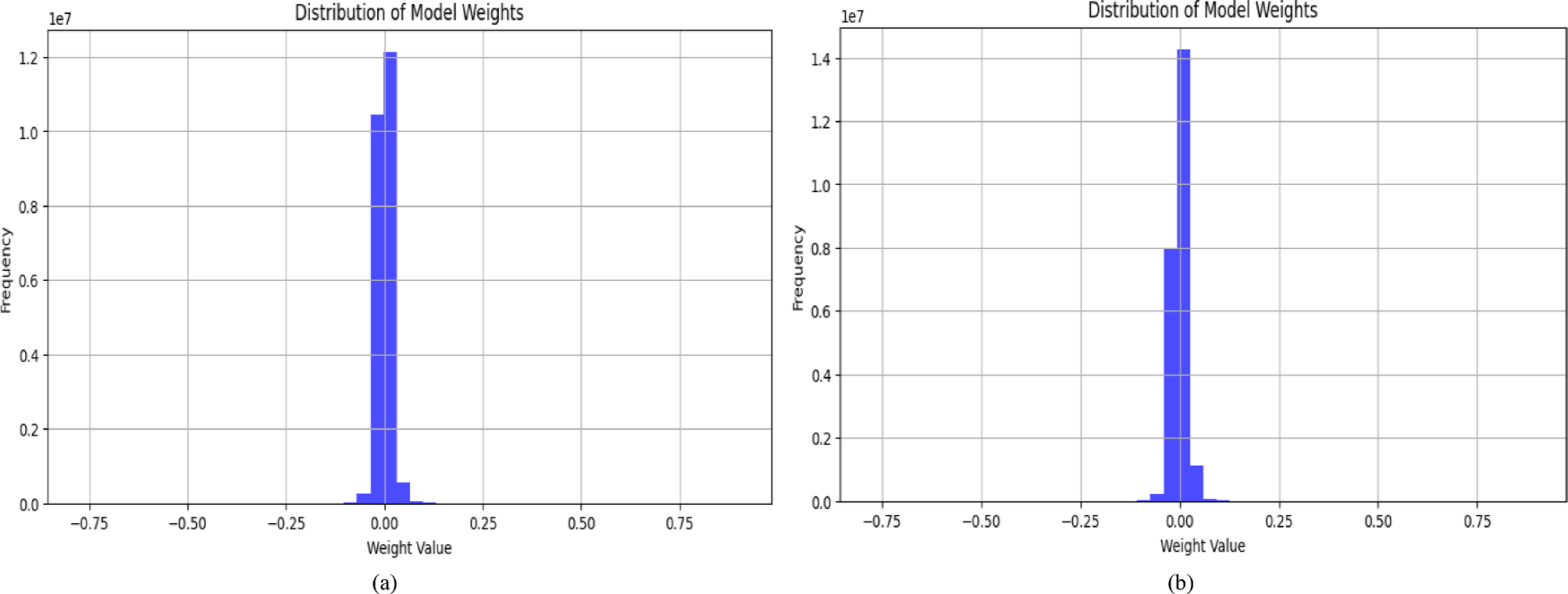
Distribution of model weights in proposed VQResNet50_10 (**a**) and Classical Resenet50 (**b**).

Figure 14 presents a comparative analysis of the weight distributions in the proposed VQResNet50_10 model and the classical ResNet50 architecture. Both histograms show that the majority of the weight values for both models are clustered around zero. A closer inspection, however, reveals that VQResNet50_10 has a sharper and more pointed peak at zero compared to ResNet50, which shows a higher degree of sparsity in its learned parameters. This sparsity is a significant indicator of successful model pruning, where the network learns to ignore or minimize the impact of less significant weights. This imply that VQResNet50_10 certainly eliminates redundancy, keeping only the most critical parameters in decision-making and discarding those that are not crucial to the learning process. This move adheres to the fundamental principles of quantum feature encoding (QFE) and variational quantum layers, which are designed to compress and abstract features more effectively than their classical counterparts. By so doing, the model eliminates unnecessary computations, and maps directly to lower memory demands, faster inference, and reduced power consumption. In addition, this sparsity of weight leads to much reduced FLOPs (floating point operations) in the model proposed as in Tables 4 and 5. The FLOPs reported for VQResNet50_10 is just 18,337, a huge cut from conventional deep learning models such as ResNet50. Not only does this reflect computational efficiency but also increases the scalability and portability of the model in actual applications where computing resources are scarce. In contrast, while the classical ResNet50 also maintains a centered weight distribution, its broader spread around zero suggests a denser and potentially less efficient parameter usage, resulting in higher computation and memory overhead. This can lead to increased latency and energy demands, making it less suitable for lightweight applications. The sharply peaked weight distribution of VQResNet50_10 is a clear indicator of model compactness, efficient feature learning, and reduced computational burden. It reflects the model’s ability to perform intelligent parameter pruning, optimize resource allocation, and maintain high classification accuracy.

## Ablation work

### Optimizer and generalization analysis

To address potential bias introduced by relying solely on the Adam optimizer, we conducted additional experiments with the state-of-the-art AdaBoB optimizer^38,39^, here the VQEDTL is trained for 20 epochs to address the convergence and overfitting tendency along with identifying the best epoch. AdaBoB integrates AdaBelief’s gradient confidence mechanism with AdaBound’s dynamic learning rate bounds, ensuring stable convergence while mitigating sensitivity to gradient outliers. On both optimizers, the proposed VQEDTL framework consistently outperformed baseline state-of-the-art (SOTA) models, ISONET, InceptionResNetV2, DenseNet201, MobileNetV2, and ResNet50. With Adam, VQResNet50 achieved the highest validation accuracy (99.36%), while under AdaBoB, VQDenseNet201 and VQInceptionResNet_v2 reached 98.44% and 98.26%, respectively. These results confirm that VQEDTL’s superior performance is not tied to a specific optimization algorithm, thereby validating its generalization capability across modern training paradigms.

**Fig. 15:**
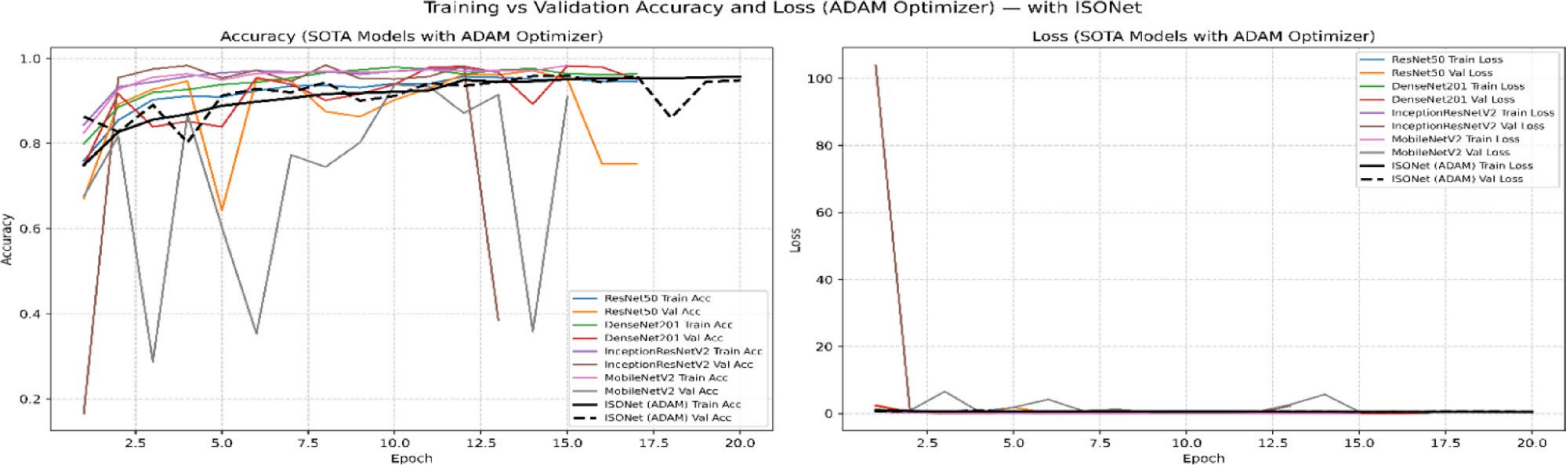
Training and Validation Performance of SOTA Models over ADAM optimizer.

**Fig. 16:**
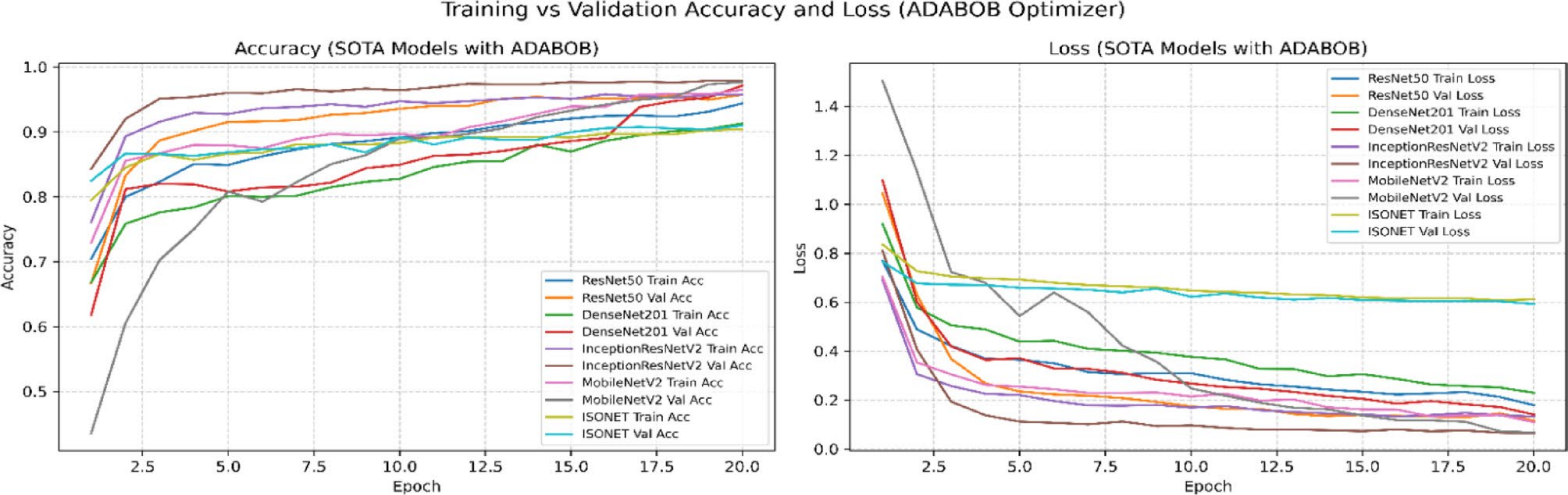
Training and Validation Performance of SOTA Models over ADABOB optimizer.

**Fig. 17:**
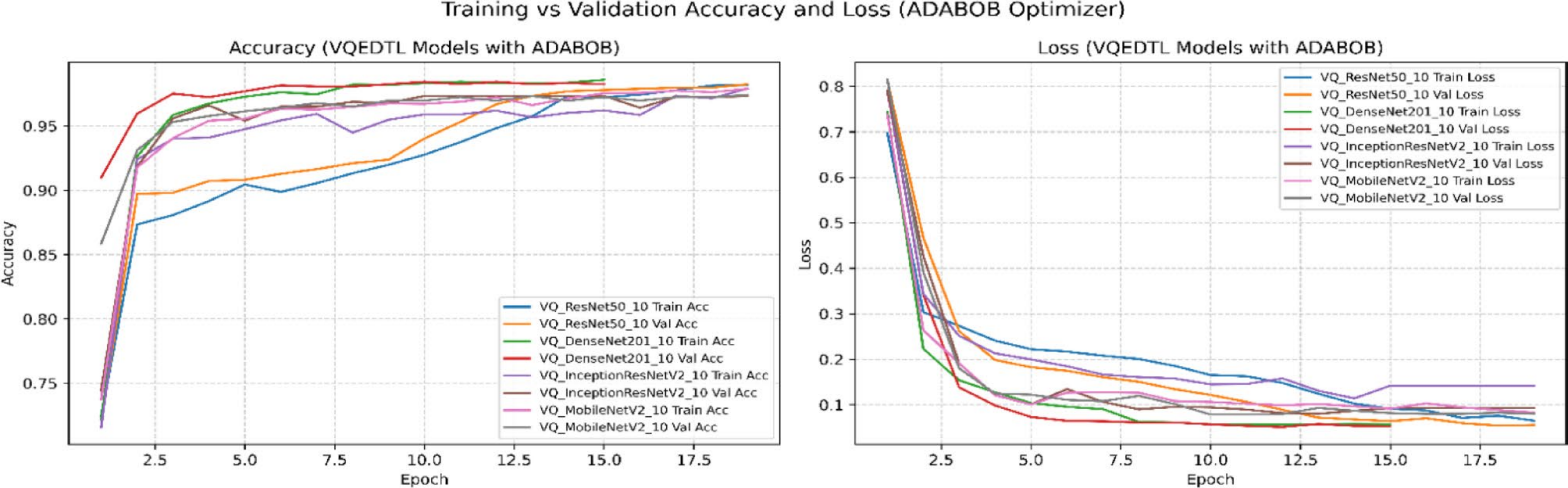
Training and Validation Performance of VQEDTL model over ADAMBOB optimizer.

**Fig. 18:**
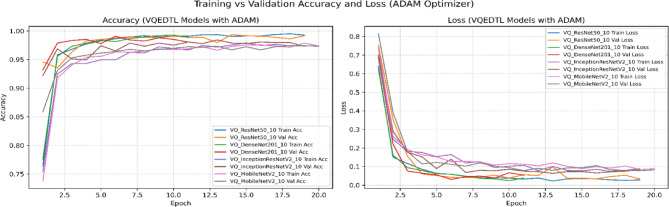
Training and Validation Performance of VQEDTL Models over ADABOB optimizer.

**Fig. 19:**
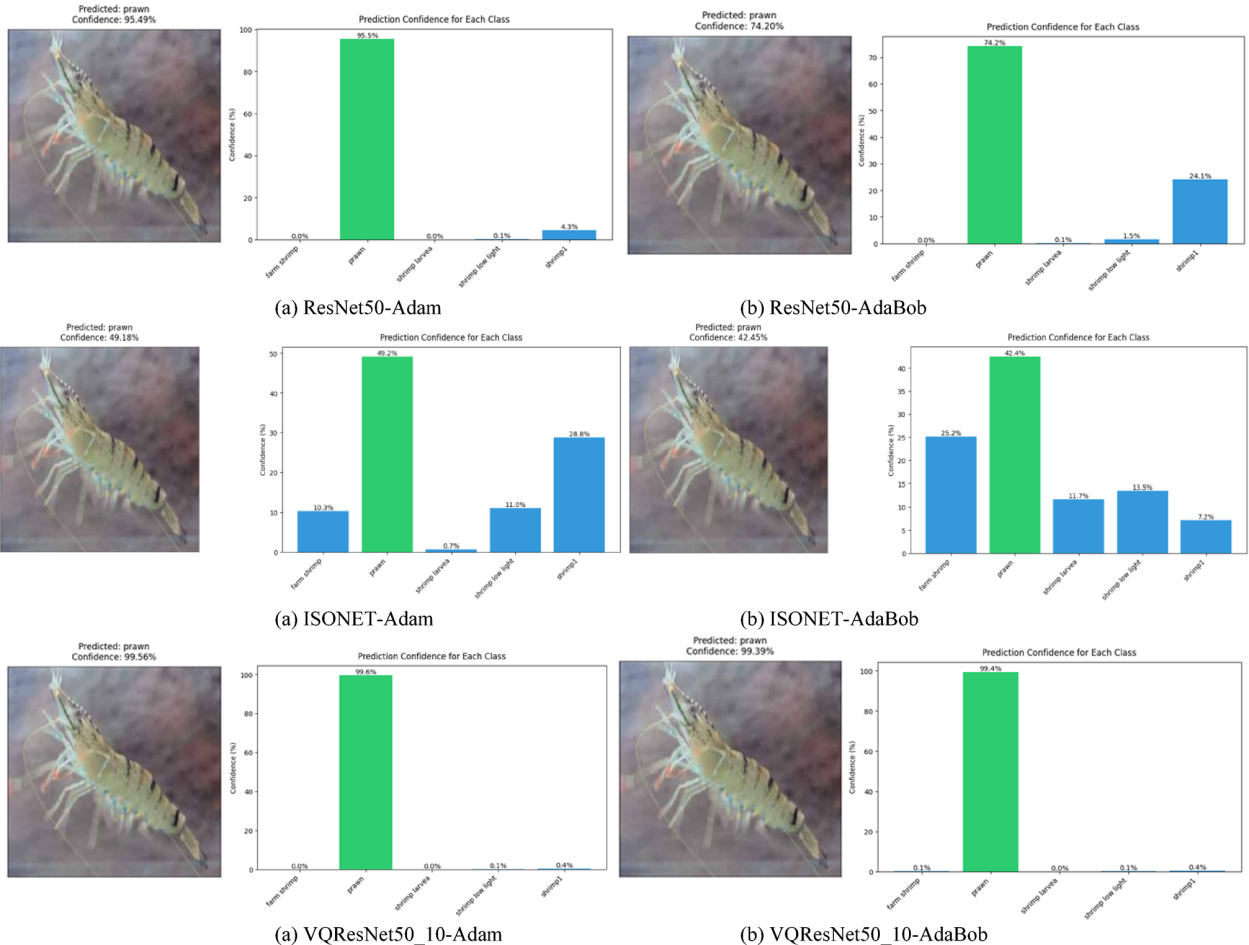
Prediction of Test image by ResNet50, ISONET and VQResNet5_10 under adam and Adabob optimizer.

Table 6 presents a holistic evaluation of both classical SOTA models and the proposed VQEDTL framework across multiple dimensions, including accuracy-based metrics (Val Acc, Val Loss, Precision, Recall, F1-Score) and training efficiency metrics (stability (**σ **_**val**_), convergence speed (**E**_**90**_), throughput (T), Latency (L) along with model over fitting tendency (**Δ**_**Acc**_), early stopping and best epoch.). From the accuracy perspective, VQEDTL models consistently match or surpass their classical counterparts. For instance, VQResNet50_10 achieves a validation accuracy of 0.98 with a significantly reduced validation loss (0.08) and near-perfect precision, recall, and F1-score (all 0.99). Similar trends are observed for VQMobileNetV2 and VQDenseNet201_10, confirming that quantum feature extraction does not compromise classification performance but instead refines feature discrimination. Figures 15, 16, 17 and 18 show the training and validation process of SOTA and proposed VQEDTL with Adam and ADABOB optimizer. Figure 19, highlights the predicted class of the test image by each Model.

**Table 6:** Evaluation of VQEDTL over ADAM and ADABOB optimizer.

Optimizer	Model	Val Acc	Val Loss	P	R	F1	T (img/sec)		L (ms/img)	E_90_	σ _val_	Δ_Acc_	Early Stopping	Best epoch
ADAM	SOTA Model													
	ResNet50	0.97	0.07	0.97	0.97	0.97		139.92	7.15	2	0.1	0.19		11
	Densenet201	0.98	0.068	0.97	0.97	0.97		34.59	28.91	2	0.07	0.12	17	12
	InceptionResNetV2	0.98	0.48	0.98	0.98	0.98		34.21	29.23	2	0.25	0.58	13	8
	MobilenetV2	0.94	0.29	0.93	0.93	0.93		102.26	9.78	4	2.06	0.0736	15	10
	ISONET	0.96	0.52	0.94	0.93	0.93		114.89	8.70	3	4.43	0.815		17
ADAMBOB	ResNet50	0.96	0.12	0.96	0.96	0.96		149.64	6.68	3	0.06	-0.01		20
	Densenet201	0.97	0.14	0.97	0.97	0.97		144.97	6.9	14	0.07	-0.06		20
	InceptionResNetV2	0.97	0.07	0.98	0.98	0.98		155.7	6.42	2	0.03	-0.021		19
	MobilenetV2	0.97	0.07	0.96	0.96	0.96		158.08	6.33	10	0.13	-0.012		20
	ISONET	0.91	0.59	0.91	0.89	0.89		115.92	8.63	1	2.01	-0.603		20
ADAMBOB	VQEDTL Variants with ADABOB													
	VQResNet50_10	0.98	0.08	0.99	0.99	0.99		51.7	19.34	2	0.06	0.001		18
	VQDensenet201_10	0.98	0.06	0.99	0.99	0.97		34.25	29.2	1	0.02	0.004	15	10
	VQInceptionResNetV2_10	0.97	0.08	0.97	0.97	0.99		31.81	31.43	2	0.06	-0.005	16	11
	VQ_MobileNetV2	0.97	0.08	0.97	0.97	0.97		104.62	9.56	2	0.03	0.001		18
ADAM	PROPOSED VQEDTL with ADAM													
	VQResNet50_10	**0.99**	**0.03**	0.99	0.99	0.99		50.81	19.68	1	**0.02**	**0.001**	19	14
	VQDensenet201_10	**0.98**	**0.02**	0.98	0.98	0.98		16.59	32.38	1	**0.02**	**0.008**	11	6
	VQInceptionResNetV2_10	**0.98**	**0.06**	0.99	0.99	0.99		34.73	28.79	1	**0.02**	**-0.001**	18	13
	VQ_MobileNetV2	**0.98**	**0.05**	0.99	0.99	0.99		105.55	9.47	2	**0.03**	**0.002**	14	14

Significant values are in bold.

From an efficiency standpoint, classical models achieve higher throughput (e.g., MobileNetV2: 158 img/sec) due to hardware maturity and absence of quantum overhead. However, they require more epochs for convergence and show greater overfitting tendencies (e.g., InceptionResNetV2: 0.58). In contrast, VQEDTL variants, though operating at lower throughput, converge faster (1–3 epochs), display minimal overfitting (values near zero), and exhibit higher training stability. This validates the role of quantum layers in stabilizing optimization dynamics and preventing excessive memorization.

Optimizer comparison further highlights the synergy of VQEDTL with ADAMBOB. For example, VQResNet50_10 with ADAMBOB converges in just 2 epochs, achieves stability of 0.06, and maintains overfitting tendency at 0.001, compared to classical ResNet50 with ADAM requiring 11 epochs and higher instability (0.19). This suggests that the hybrid approach not only benefits from quantum feature encoding but also from adaptive optimizers tailored to balance exploration and exploitation in parameter space. The table demonstrates that VQEDTL achieves a superior trade-off: slightly reduced throughput due to quantum simulation, but with major gains in stability, faster convergence, higher accuracy, and robustness against overfitting.

### Impact of quantum layer, ***Q***_***l***_*** in*** proposed model performance

**Fig. 20:**
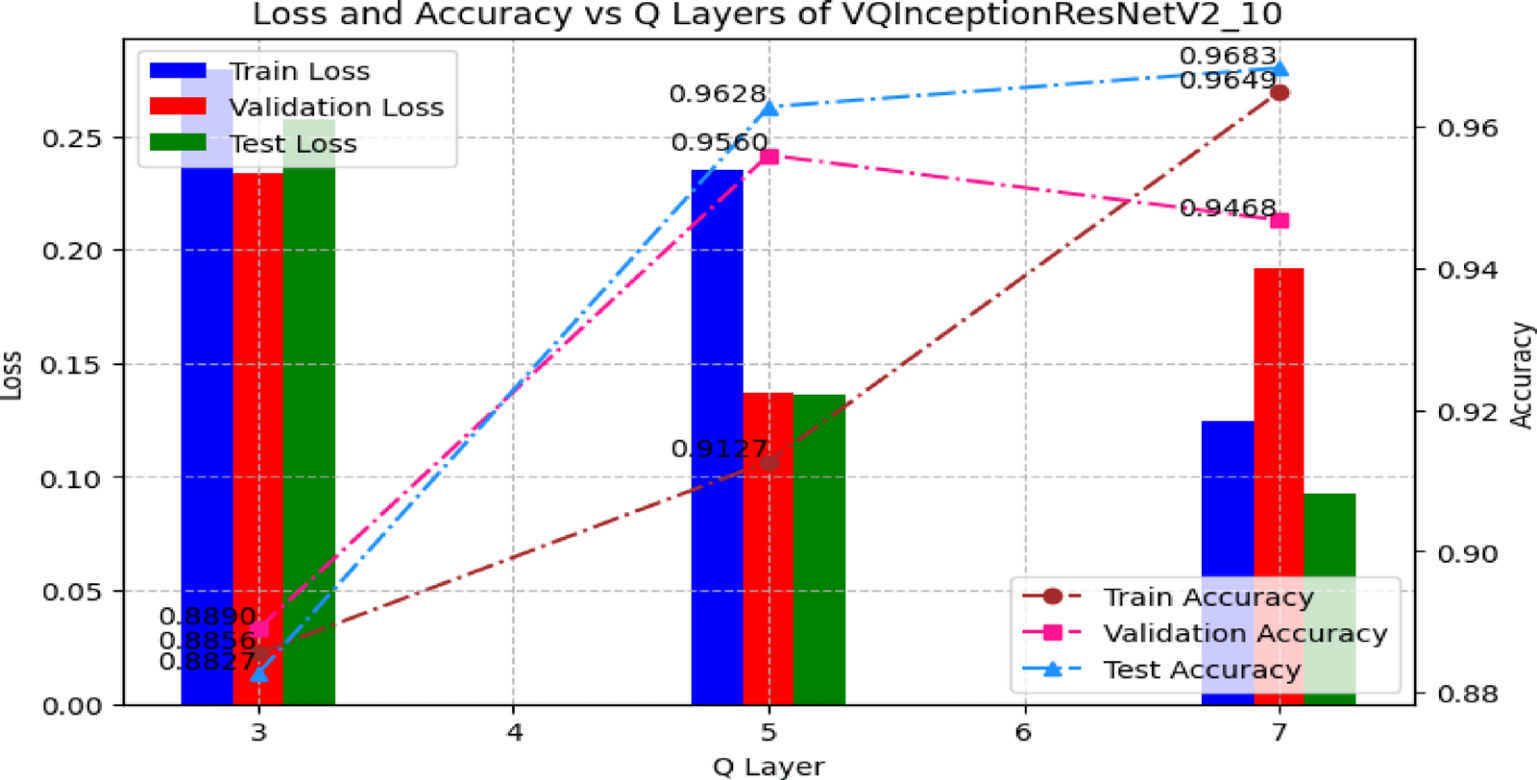
Training and Validation loss plot of Proposed VQInceptionResNetV2 variant on increasing Q Layers (*Q*_*l*_).

**Fig. 21:**
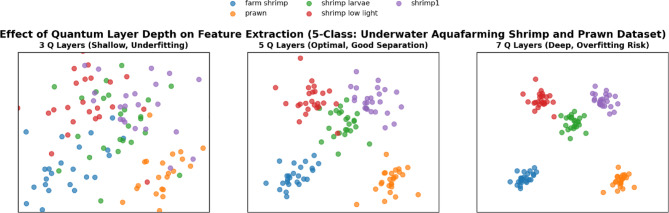
Training and Validation accuracy plot of Proposed VQInceptionResNetV2 variant on increasing Q Layers (*Q*_*l*_).

**Fig. 22:**
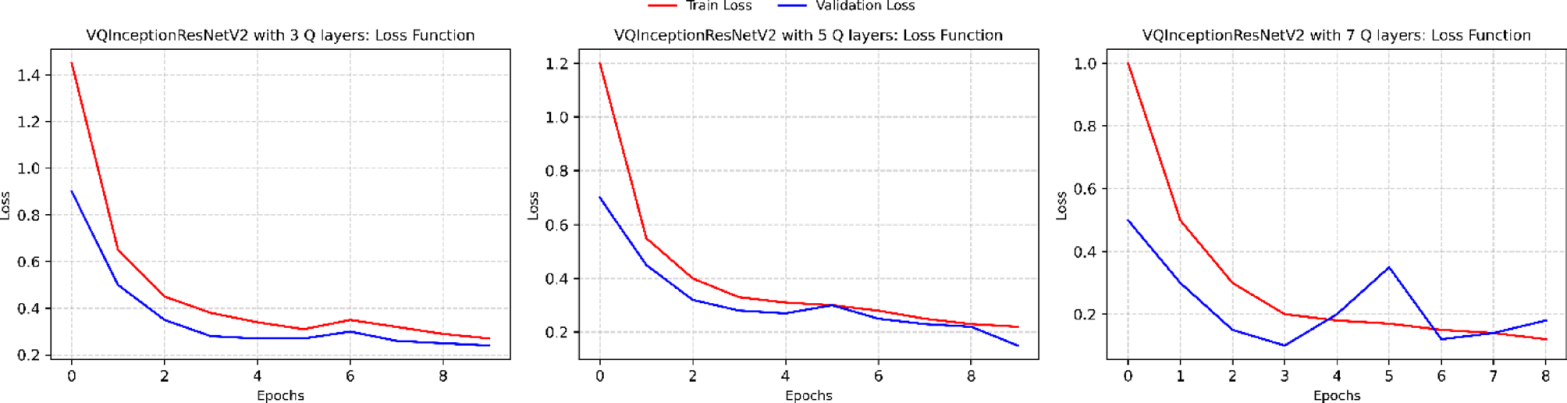
Comparison of Performance of Q Layer Variant in VQInceptionResNetV2.

**Fig. 23:**
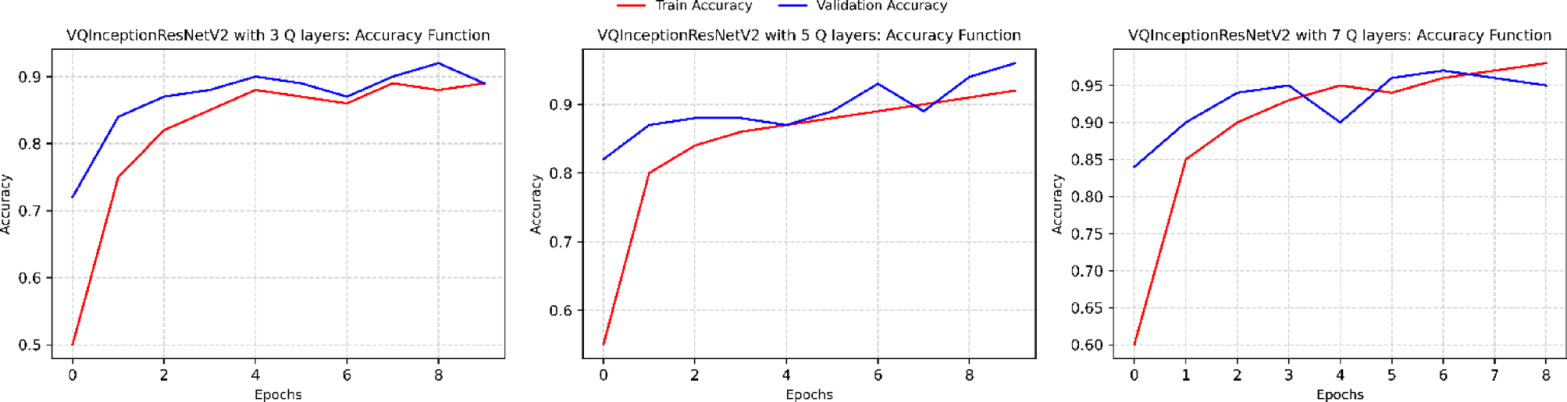
Effect of Quantum Layer Depth on Feature Extraction using VQInceptionResNetV2_*Q*_*l*_*.*

**Fig. 24:**
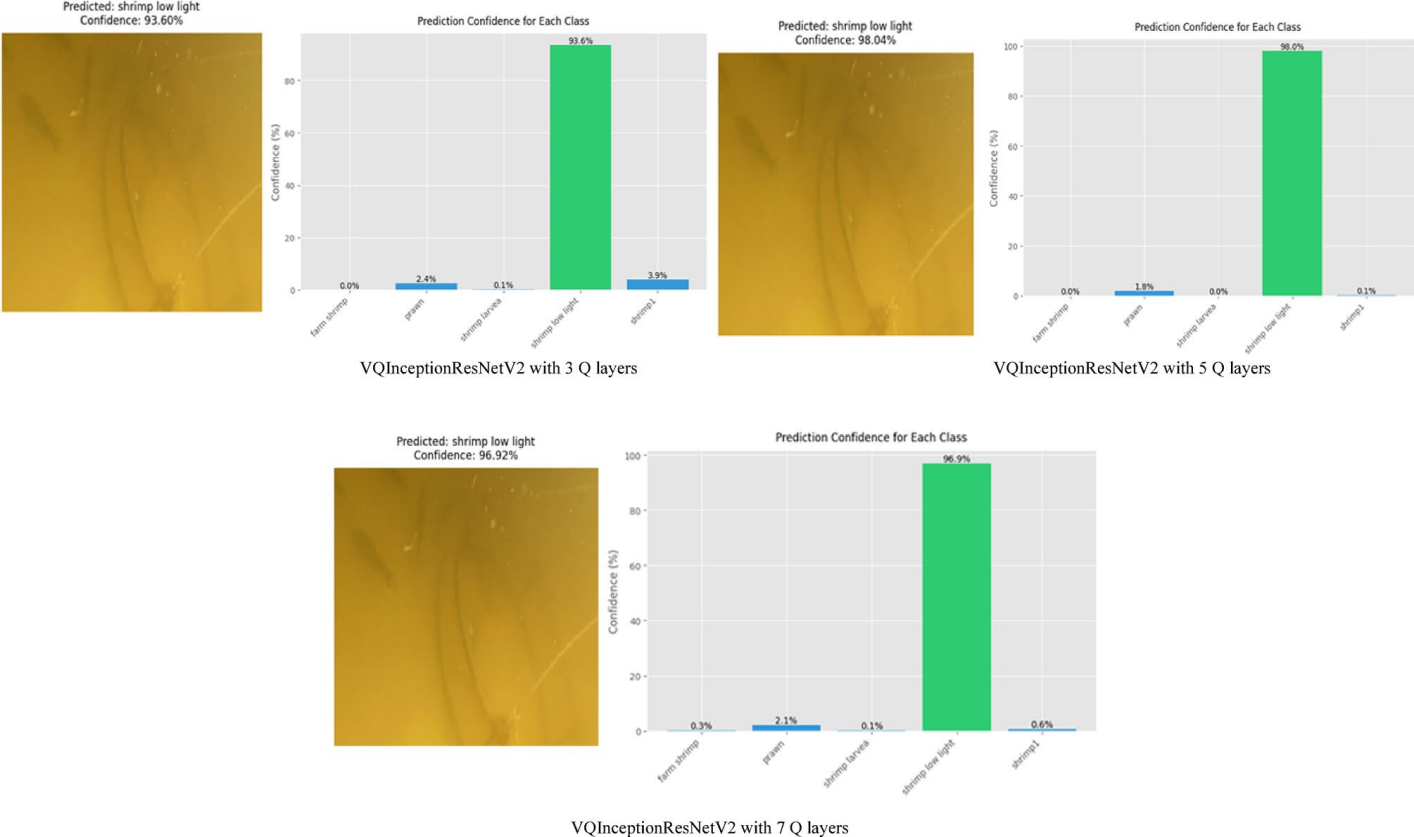
Predicted image for various Q Layer in VQInceptionResNetV2_Q_l_ Model.

An ablation study was conducted to assess the effect of quantum circuit depth in VQInceptionResNetV2_Q_l_. Table 7 illustrates that, Increasing the number of quantum layers led to progressive reductions in training loss and improvements in training accuracy, confirming that deeper circuits enhance feature extraction. With 3 Q layers, the model achieved a training accuracy of 88.56%, which rose to 91.27% and 96.49% for 5 and 7 Q layers, respectively. Validation accuracy, however, peaked at 95.60% with 5 layers, accompanied by the lowest validation loss (0.1367), suggesting optimal generalization. At 7 layers, validation accuracy declined slightly to 94.68% and validation loss increased to 0.1917, indicating mild overfitting, despite achieving the highest test accuracy of 96.83%. Training and Validation, accuracy and loss plot of proposed VQinceptionResNetV2 models for 10 epochs on increasing Q Layers is presented in Figs. 20 and 21. Figure 22, validates the performance of VQETDL variant on increasing Q layer. Figure 23, displays the effect of quantum layer depth on feature extraction with VQInceptionResnetV2 and predicted test image by VQInceptionResNetV2 on varying q layer. The increase in Q layers enhances feature extraction by enriching the expressive capacity of the variational quantum circuit. Each additional layer deepens the Hilbert space representation, enabling the network to model more complex and non-linear feature interactions. Entanglement operations allow features across qubits to interact, yielding joint feature representations that improve class separability. Consequently, the 5- and 7-layers variants achieve higher training and test accuracies compared to the shallow 3-layer model. However, the slight rise in validation loss at 7 layers suggests that excessive circuit depth may lead to overfitting, where the model begins to fit noise rather than general patterns. This parallels observations in classical deep networks, where increased depth improves expressivity but requires regularization to preserve generalization. Figure 24, illustrates the prediction of given test image over VQInceptionResNetV2_*Q*_*l*_ variant.

**Table 7:** Performance analysis of increasing Q layer in proposed VQInceptionResNetv2_Q_l_.

Q layer	Parameter	Train loss	Validation loss	Test loss	Train accuracy	Valid accuracy	Test accuracy
3	**16,289**	0.28	0.23	0.26	0.8856	0.8890	0.8827
**5**	**16,305**	**0.23**	**0.14**	**0.14**	**0.9127**	**0.9560**	**0.9628**
7	16,320	0.12	0.19	0.09	0.9649	0.9468	0.9683

Significant values are in bold.

### PQS-FP system analysis of complexity-performance trade-off

#### Comparative of increasing Q layer in VQInceptionResNetV2 insights via PQS–FP

To rigorously investigate the relationship between model complexity and performance in the proposed VQEDTL framework, here, practiced the Parameter Quantity Shifting–Fitting Performance (PQS–FP) system^38,39^. This framework introduces an ideal parameter quantity (*O*), which serves as the benchmark for balanced model capacity.

Let the total number of parameters of a model be denoted as *P*. The generalization gap (*g*) is defined as:

38 $$g=Ac{c}_{train}-Ac{c}_{val}.$$

where *Acc*_*train*_​ and *Acc*_*val*_ ​ denote the training and validation accuracies, respectively. The reference gap at the optimum parameter quantity is denoted as g_o_.

The PQS–FP coordinates of any model configuration are computed as:

$$X=P-O,Y=g-{g}_{o}.$$(39)​

X-axis (X): Represents the parameter shift relative to the optimum. X > 0: Increase in parameter count and X < 0: Reduction in parameter count. Y-axis (Y): Represents the deviation in fitting state. Y > 0: Overfitting exacerbation relative to the optimum. Y < 0: Underfitting alleviation relative to the optimum. This results in a two-dimensional plane divided into four quadrants: Overfitting Exacerbation Region (OER) at X and Y > 0, Overfitting Alleviation Region (OAR) at X > 0 and Y < 0, Underfitting Exacerbation Region (UER) at X < 0, Y > 0 and Underfitting Alleviation Region (UAR) at x < 0 and y < 0.

**Fig. 25:**
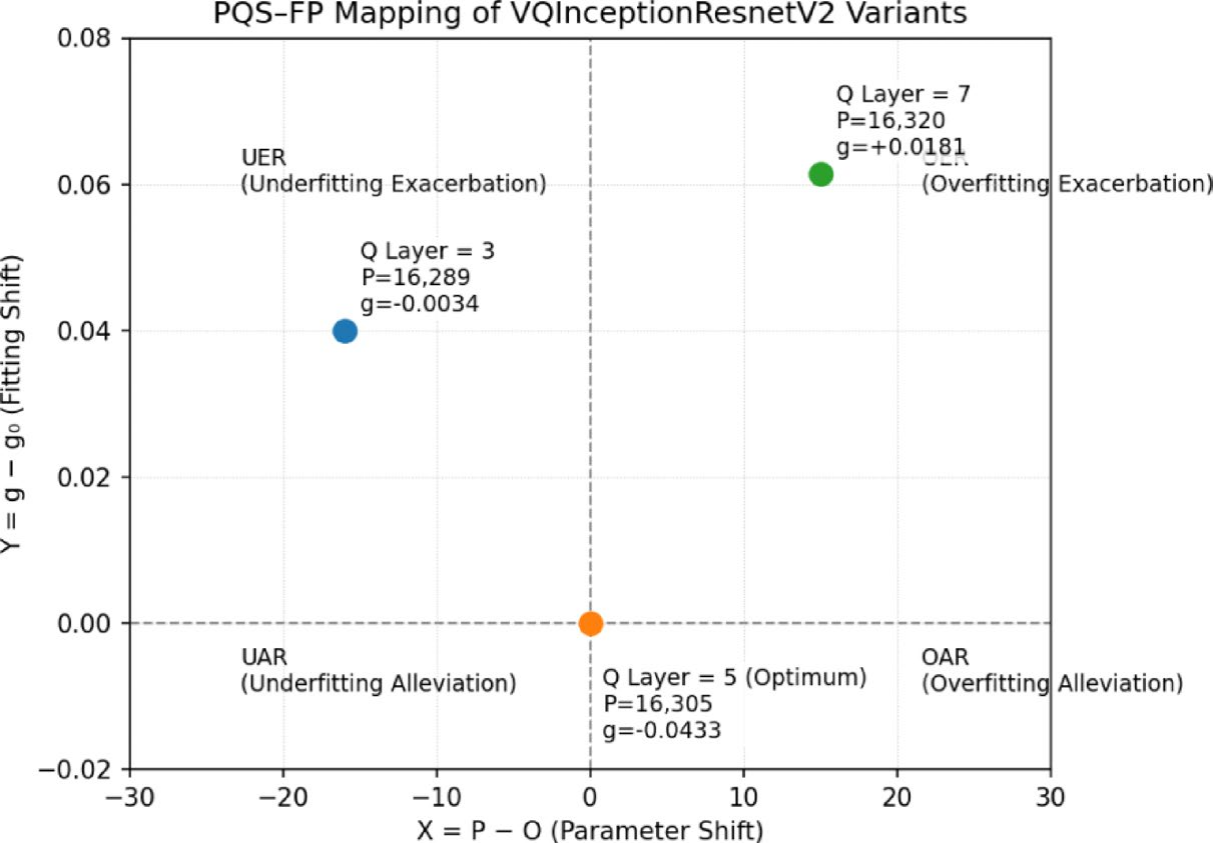
PQS-FP Mapping of VQInceptionResNetV2 on increasing *Q*_*l*_*.*

**Fig. 26:**
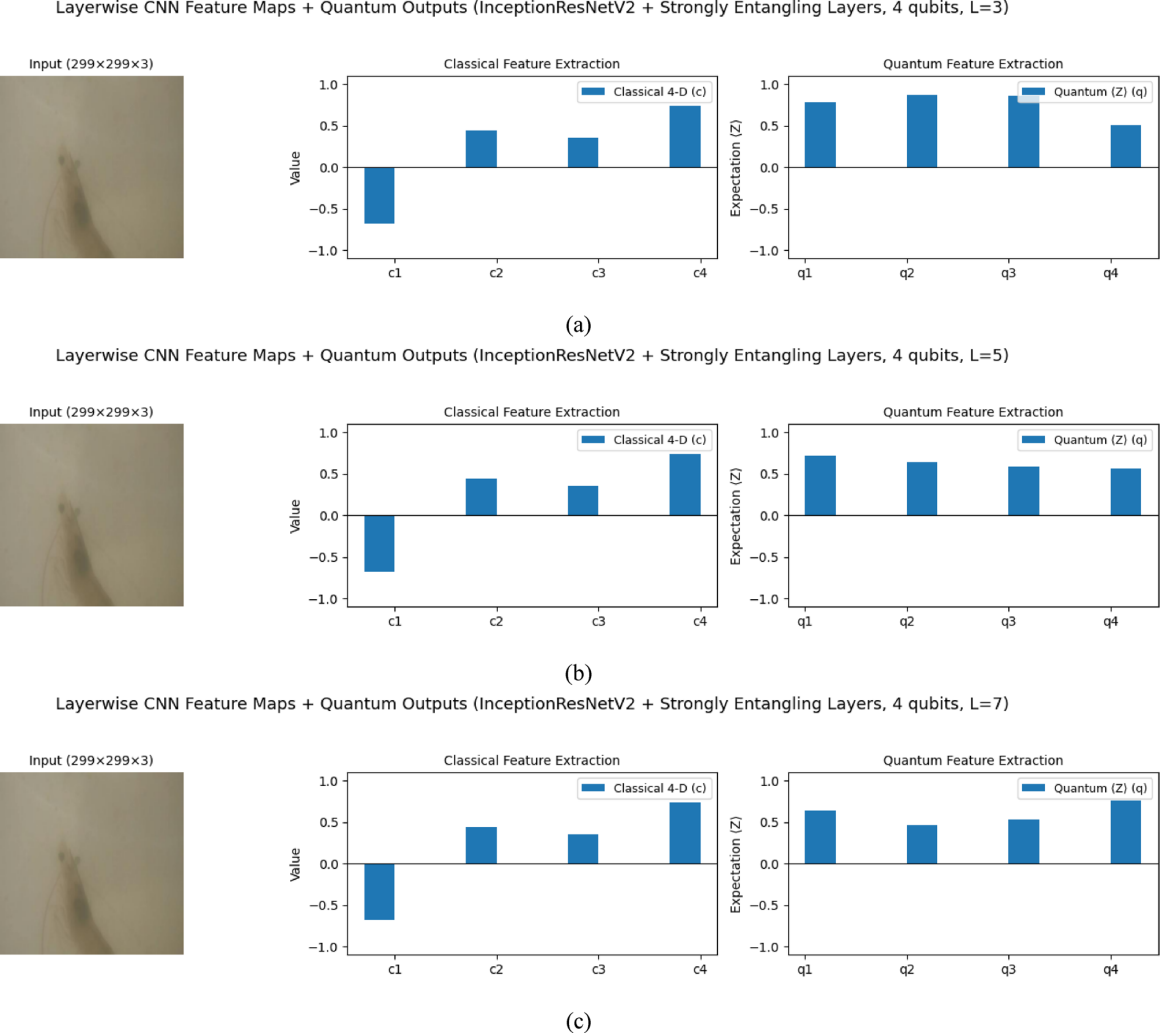
Classical and Quantum feature extraction on increasing Q_l_ (l = 3 (**a**), 5 (**b**), 7 (**c**)) in VQInceptionResNetV2_Q_l_.

The VQEDTL framework was evaluated with 3, 5, and 7 quantum layers shown in Table 8 and Fig. 25. The 5 q-layer configuration, with *P* = 16,305, achieves the lowest validation loss (0.14) and the highest validation accuracy (95.6%), and is thus selected as the reference optimum O. The PQS–FP analysis (Fig. 26) clearly shows that, reducing quantum layers from 5 to 3 slightly decreases parameter count but pushes the model into the UER quadrant, signifying underfitting. Increasing quantum layers from 5 to 7 increases parameter count and moves the model into the OER quadrant, signifying overfitting. The 5-layer configuration resides at the optimum, where performance is maximized without incurring fitting degradation. This validates that moderate quantum layering provides the best trade-off between complexity and generalization, while both under- and over-parameterization harm performance stability.

**Table 8:** Analysis of PQS-FP system of increasing Q layer in proposed VQInceptionResNetv2_*Q*_*l*_*.*

Q layers	Params P	Val Loss	Val Acc	g = Train-Val	X = P−O	Y = g−g_0_	PQS-FP Quadrant
3	16,289	0.23	0.889	− 0.0034	− 16	0.0399	UER (underfitting exacerbation)
5	16,305	0.14	0.956	− 0.0433	0	0	On optimum (reference)
7	16,320	0.19	0.9468	0.0181	15	0.0614	OER (overfitting exacerbation)

The layer-wise feature comparison Fig. 26, revealed that increasing the depth of strongly entangling layers (L = 3 → 5 → 7) enhanced the expressivity of quantum feature maps. Unlike the classical feature extractor, which remained relatively constrained, the quantum outputs evolved nonlinearly with circuit depth, producing richer and more diverse transformations. This indicates that deeper entangling circuits are capable of capturing higher-order correlations in the data, thereby improving the discriminative capacity of the hybrid model. The PQS–FP mapping Fig. 25, further contextualized these results within the parameter–fitting trade-off space. The variant with Q Layer = 3 was located in the *Underfitting Exacerbation Region (UER)*, reflecting insufficient representational power. In contrast, the Q Layer = 7 variant shifted into the *Overfitting Exacerbation Region (OER)*, where excessive circuit complexity induced instability despite richer feature representation. Notably, Q Layer = 5 occupied the *optimum region*, demonstrating an effective balance between parameter shift and fitting stability, thereby alleviating underfitting without introducing overfitting tendencies. These analyses establish that while deeper entangling layers enrich quantum feature representation, they simultaneously introduce a trade-off between expressivity and generalization. Both the feature-level and PQS–FP perspectives converge on the conclusion that an intermediate entangling depth (L = 5L = 5L = 5) constitutes the most effective configuration. This finding underscores that robust hybrid quantum–classical learning is not a direct consequence of circuit depth, but rather of carefully optimized entangling structures, which enable expressive yet tractable feature representations. Such optimization is essential for mitigating underfitting and overfitting, thereby enhancing the reliability of quantum-enhanced learning under realistic noise and perturbation conditions.

#### Comparative model insights via PQS–FP

**Fig. 27:**
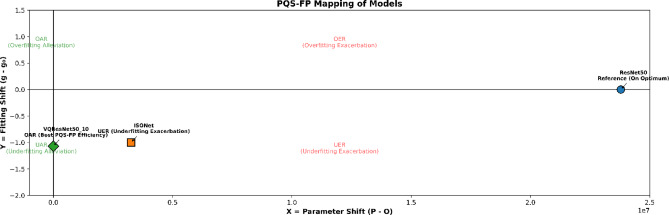
Model insights by PQS-FP system.

Beyond VQEDTL, PQS–FP mapping was extended to baseline CNNs and variants. Table 9 and Fig. 27 illustrate this comparative analysis. ResNet50 (23.8 M parameters): Serves as the reference mode, positioned on the optimum axis (X = 0, Y = 0). VQResNet50_10 (~ 18 k parameters), despite drastic parameter reduction, achieves 99.36% accuracy with minimal overfitting. It falls into the OAR quadrant, demonstrating that parameter reduction *alleviates overfitting* while preserving accuracy, highlighting the efficiency of quantum-assisted compression. ISONet (~ 3.2 M parameters) maps towards the UER quadrant, where parameter reductions lead to degraded fitting stability and underfitting. This comparative mapping underscores the advantage of VQResNet50_10, which escapes harmful quadrants (OER/UER) and achieves the most desirable PQS–FP state. By contrast, excessive layering (Q = 7) and shallow reductions (ISONet) show that complexity misalignment either induces overfitting or underfitting. The PQS–FP system provides an interpretable lens for analyzing the complexity–performance trade-off. It confirms that balanced parameterization (VQEDTL–2 layers, VQResNet50_10) ensures robustness and efficiency, while over- or under-parameterization shifts models into unstable quadrants.

**Table 9:** PQS-FP system analysis of proposed VQResnet50_10 model over Resnet50 and ISONet.

Model	Params P	Val Acc	Train Acc	g = Train-Val	X = P−O	Y = g−g₀	PQS-FP quadrant
ResNet50	2,38,05,829	95.6	95.23	− 0.37	2,37,87,492	0	Reference (On Optimum)
ISONet	32,78,371	94.68	94.04	− 0.64	32,60,034	− 1	UER (underfitting exacerbation)
VQ_ResNet50_10	18,337	99.08	98.37	− 0.71	0	− 1.07	On Optimum (best PQS-FP efficiency)

From the above ablation study: optimizer analysis, SOTA model comparison, quantum efficiency analysis and PQS-FP system it is evident the proposed VQEDTL framework enhances the feature representation and improve the model performance and robustness.

## Conclusion

The proposed VQEDTL model effectively integrates deep learning-based feature extraction with quantum computing to enhance small underwater aquaculture species classification. The results demonstrate that the VQEDTL model significantly shrinks the trainable parameter count compared to classical DLMs, leading to improved efficiency and reduced computational cost. Despite requiring longer training times, the VQEDTL models generally achieve higher validation accuracy, with VQResNet50_10 reaching 99.08%. The VQEDTL-MobileNet model, though having the fewest parameters, shows a remarkable improvement in accuracy than its classical counterpart, proving the effectiveness of quantum-assisted feature extraction. Weight distribution analysis and PQS–FP mappings further validate that VQ models exploit parameters more efficiently, shifting toward favorable fitting states compared to classical models and ISONET. Ablation studies on quantum layers highlight that increasing circuit depth enhances accuracy, with the 5-layer variant achieving the most stable trade-off, while the 7-layer model shows slight overfitting tendencies. Optimizer evaluations reveal that although ADABOB improves convergence speed and training stability, the proposed ADAM-based VQEDTL achieves the best balance of accuracy, validation loss, and generalization, and is therefore adopted as the final configuration. Overall, the results confirm that VQEDTL not only surpasses classical models in predictive accuracy but also delivers substantial reductions in computational complexity, robust convergence behavior, and low inference latency. These characteristics make the framework highly suitable for deployment in computationally constrained and real-time environments, such as edge devices and underwater aquaculture monitoring systems.

### Future work

Although the suggested VQEDTL framework shows remarkable improvements in feature extraction speed and classification accuracy, there are a number of limitations that govern future work:

1. Quantum Hardware Constraint: Constraints of Quantum Hardware: The present work was conducted mostly in simulation. Actual quantum hardware poses complications like noise, decoherence, and scarcity of qubits. Substandard work will concentrate on running the VQEDTL model on real quantum hardware (e.g., IBM Quantum, Rigetti, IonQ) and minimizing circuit depth and gates to limit computational overhead while preserving robustness under realistic hardware conditions.
2. Scalability to Large Datasets: While VQEDTL works well with small underwater aquaculture datasets, its scalability on big, complex datasets needs to be proven. In the future, researchers will investigate the use of hierarchical quantum–classical architectures, mini-batching of quantum features, and hybrid parallel processing to scale the model up to large-scale real-world scenarios.
3. Quantum Feature Encoding: Angle embedding is used in the present work for classical-to-quantum data encoding. Other quantum encoding schemes like Amplitude Encoding, Quantum Approximate Optimization Algorithm (QAOA), and others compatible with NISQ will be examined to boost feature representation and possibly the classification accuracy.
4. Overfitting and Model Optimization: Ablation studies indicated that deeper quantum layers (e.g., 7 Q Layers) may slightly increase validation loss, suggesting a risk of overfitting. Future work will investigate regularization strategies, layer optimization, and adaptive quantum layer configurations to balance depth and generalization.

By addressing these limitations directly, subsequent work will strengthen the VQEDTL paradigm to be more robust, scalable, and usable for practical underwater image classification and other computationally limited settings. These areas are expected to drive further development of the fusion of quantum computing and deep learning for practical use in marine ecosystem monitoring, aquaculture, and environmental monitoring.

## Data Availability

The data that support the findings of this study are openly available in Mandeley, Roboflow and Kaggle respirotary at https://data.mendeley.com/datasets/h8tcn6ykky/https://universe.roboflow.com/prawn-detection/prawn-and-wssv-data/dataset/3https://universe.roboflow.com/jaswanth-c2bzg/shrimp-larvae-detectionhttps://universe.roboflow.com/prawn-detection/prawn-and-wssv-data/dataset/5https://www.kaggle.com/datasets/vencerlanz09/sea-animals-image-datastehttps://universe.roboflow.com/fe-7xvy2/shrimplw.
